# Applications of Ceramic/Graphene Composites and Hybrids

**DOI:** 10.3390/ma14082071

**Published:** 2021-04-20

**Authors:** Cristina Ramírez, Manuel Belmonte, Pilar Miranzo, Maria Isabel Osendi

**Affiliations:** Instituto de Cerámica y Vidrio (ICV), Consejo Superior de Investigaciones Científicas, CSIC. Kelsen 5, 28049 Madrid, Spain; mbelmonte@icv.csic.es (M.B.); pmiranzo@icv.csic.es (P.M.)

**Keywords:** ceramic graphene composites, ceramic graphene hybrids, applications, ceramic composites, graphene, graphene-related materials, graphene oxide, graphene nanoplatelets

## Abstract

Research activity on ceramic/graphene composites and hybrids has increased dramatically in the last decade. In this review, we provide an overview of recent contributions involving ceramics, graphene, and graphene-related materials (GRM, i.e., graphene oxide, reduced graphene oxide, and graphene nanoplatelets) with a primary focus on applications. We have adopted a broad scope of the term ceramics, therefore including some applications of GRM with certain metal oxides and cement-based matrices in the review. Applications of ceramic/graphene hybrids and composites cover many different areas, in particular, energy production and storage (batteries, supercapacitors, solar and fuel cells), energy harvesting, sensors and biosensors, electromagnetic interference shielding, biomaterials, thermal management (heat dissipation and heat conduction functions), engineering components, catalysts, etc. A section on ceramic/GRM composites processed by additive manufacturing methods is included due to their industrial potential and waste reduction capability. All these applications of ceramic/graphene composites and hybrids are listed and mentioned in the present review, ending with the authors’ outlook of those that seem most promising, based on the research efforts carried out in this field.

## 1. Introduction

**Figure 1:**
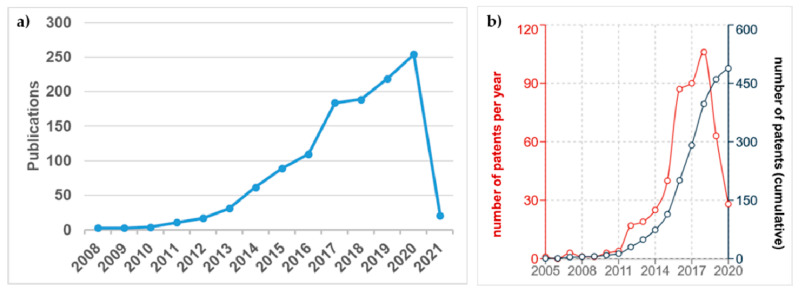
(**a**) Publications per year in the subject of ceramic/graphene composites (data collected from WOS, Clarivate Analytics); (**b**) Number of patents per year and cumulative in the same topic in either the title or the abstract (source Espacenet, EPO).

Since the monolayer isolation in 2004, graphene has been a field of incessant activity, owing to the amazing properties of graphene [1] and the scope of potential applications and uses, as stated in many articles and reviews [2,3]. Focusing on the field of ceramic/graphene composites and hybrids, the bustle relatively dims down, nevertheless, there has been permanent growth in the number of published papers since 2004 as can be seen in Figure 1a (data collected from the Web of Science (WOS) with the topic graphene + ceramic + composite). Several reviews reflect the interest in this topic, revealing the benefits of graphene and related materials (GRM)-graphene oxide (GO), reduced graphene oxide (rGO), and graphene nanoplatelets (GNP) as reinforcing agents that provide electrical and thermal transport properties or wear and friction benefits as well [3,4]. The increase in the number of documents published since 2017 is particularly noticeable, so this review aims to offer a representative description of the most relevant contributions during the 2016–2020 five-year period but concentrating on applications, which have resulted in about 340 references from a total of almost 1000 papers in the period. The applications of ceramic/graphene composites and hybrids include materials for energy production and storage, sensors, tissue engineering, electromagnetic interference shields, thermal management, thermal protection, protection against wear and corrosion, and uses in catalysis, each of which is discussed in the appropriate section, listed as follows:

2. Ceramic/graphene composites used in energy production and storage
3. Piezo and thermoelectric ceramic/graphene composites for energy harvesting
4. Sensors based on ceramic/graphene composites and hybrids
5. Ceramic/graphene composites for electromagnetic interference shielding
6. Catalytic applications of ceramic/graphene composites
7. Ceramic/graphene composites in biomedicine
8. Thermal applications of ceramic/graphene composites
9. Structural engineering applications of ceramic/graphene composites
10. Applications of additively-manufactured ceramic/graphene composites

In some cases, we have taken an ample view including inorganic materials such as metal oxides and cements, and in most cases, a slight account of the material fabrication procedure is included along with key results for the intended uses. Outcomes about composite materials fabricated by additive manufacturing methods that emphasize applications deserved a separate section because they offer interesting flexibility and potential for novel device development.

The number of patents filed in the subject (ceramic + graphene + composites) has experienced an evolution quite similar to that of research articles (Figure 1b) but obviously with fewer documents. Despite these impressive figures, commercial product development clearly falls behind, and one of the alleged reasons is the relatively high cost of GRM, with figures on the order of 1250–2500 €/kg for GO, and much lower (100–700 €/kg) for GNP [5,6], both being the most widely used products in composite fabrication. On the positive side, it is important to notice that small amounts of these GRM (typically < 10 vol.%) make a true impact on the properties of composite materials owing to their presumably high exfoliation. The main impediments for mass industrialization are linked to reliability and quality control of GRM, although some authors point the finger at the absence of a really groundbreaking application for graphene as [6]. However, favorable prospects lie ahead regarding production cost and quality control assessments for graphene materials and composites [5].

## 2. Ceramic/Graphene Composites Used in Energy Production and Storage

### 2.1. Batteries

The increasing demand for portable electronic devices and electric vehicles has generated enormous research efforts to improve specific capacity, energy density, cycling performance and safety of batteries used in energy storage systems. One of the recent strategies to address these challenges involves incorporating GRM into battery components for improved electrochemical performance due to graphene´s excellent electrical and thermal conductivities, lightweight, high specific surface area and superior mechanical strength [7]. The principal effects of adding GRM are: (i) improvement of the active material distribution, (ii) control of volumetric expansion during cycling, and (iii) boosting of diffusion processes in composite electrodes through easier electrolyte penetration between the layers and channels created. The main results are presented separately for each battery component below.

#### 2.1.1. Cathodes

Typical cathode materials are binary lithium compounds, such as LiCoO_2_, LiFePO_4_, or LiMnO_2_, exhibiting theoretical specific capacities in the range 100–300 mA·h·g^−1^, and transition metal oxides and metal chalcogenides structures in which Li-ion can be intercalated, giving specific capacitance of 100–200 mA·h·g^−1^ [8]. Different LiFePO_4_ (LFP)/graphene composites have been processed to improve LFP low electronic conductivity and slow Li transfer. For instance, Ma et al. [9] and Oh et al. [10] developed 3D graphene and rGO networks on sacrificial foams coated with LFP nanoparticles (*np*). The results indicated that graphene can reduce Li penetration pathways delivering discharge capacities of 160 mA·h·g^−1^ (at 1 C) and 131 mA·h·g^−1^ (at 2 C), respectively. When both Y_2_O_3_ and rGO were added to a LiNi_0.8_Co_0.15_Al_0.05_O_2_ compound [11], favorable effects on the compound decomposition and stabilization of the electrode-electrolyte interface were observed, as well as an improvement of the conductive network. The discharge capacity reached 191 mA·h·g^−1^ at 0.5 C with a capacity retention of 92% after 100 cycles.

The electrochemical performance of a V_2_O_5_ cathode was also improved by adding rGO for the conformation of porous composite microballs with high mechanical stability [12]. The multiple oxidation states of vanadium oxide incremented the reduction of rGO, delivering an initial discharge capacity of 375 mA·h·g^−1^ at 50 mA·g^−1^ with 80% capacity retention after 200 cycles. A 50:50 (by wt.%) composite of graphene and Co_3_O_4_ nanocatalyst promoted the oxygen reduction reaction (ORR) in a Li-air battery compared to the use of pure carbon cloth [13]; it also showed at least 30 more hours of operation than a Pt/C cathode under the same conditions and reduced the catalyst aggregation as well.

Compared to other carbon additives, the GO multilayered wrinkled structure and functional groups favored anchoring sites for particles of different size, preventing agglomeration; accordingly, well-distributed graphene sheets in between NaMnO_2_ nanorods (*nr*) showed 12% more capacity retention after 100 cycles than the material without graphene when tested in a Na-ion battery [14].

#### 2.1.2. Anodes

##### Si-Based Anodes

Due to their high specific capacity, nanostructured Si-based materials and transition metal oxides (TMO) stand out as promising alternatives to replace graphite, the most commonly used material in lithium-ion battery (LIB) anodes; however, they suffer large volume expansions during lithiation, leading to pulverization, delamination and electrode instability [15]. The wrapping of Si *np* by rGO or pristine graphene has been shown to be effective for the alleviation of Si volume expansion, protecting Si *np* from pulverization and stabilizing the solid electrolyte interface. In the case of a Si/N-doped rGO composite [16], the electrode thickness only augmented 21% after 100 cycles compared to the 76% of Si *np* electrode. The better structural behavior and improved charge transport achieved through N-doping delivered an initial discharge capacity of 1712.2 mA·h·g^−1^ with initial Coulombic efficiency of 71.5%, and reversible capacity of 1007 mA·h·g^−1^ after 50 cycles, 150% higher than Si *np*. A double coating of Al_2_O_3_ and graphene on porous Si created multipoint electrical contacts for faster charge transport and also provided mechanical integrity after 600 cycles, while maintained a capacity of 966 mA·h·g^−1^ at a current density of 1 A·g^−1^ [17]. A similar electrode, composed of nanospheres with Si core and Si_3_N_4_/graphene shell [18] showed a specific capacity of 2709 mA·h·g^−1^ with an initial Coulombic efficiency of 80%; whereas the discharge capacity was 827 mA·h·g^−1^ after 450 cycles at a current density of 2 A·g^−1^. The SiBCN/graphene composite anode derived from polyborosilazane was reported to have four times improved discharge capacity at the first cycle and seven times increased after 30 cycles compared to the material without graphene [19]. Graphene layers refined the SiBCN particle size in one order of magnitude and provided Li-ion with additional transformation sites in between the layers and at the interface with Si_3_N_4_, SiC and BNC clusters. In an electrode of SiBCN with N–S dual-doped graphene [20], increased conductivity and improved contact with the current collector were referred to, besides the creation of new active sites for Li-ion insertion. After 800 cycles, it delivered 785 mA·h·g^−1^ (at a current density of 450 mA·g^−1^) with 99% Coulombic efficiency.

**Figure 2:**
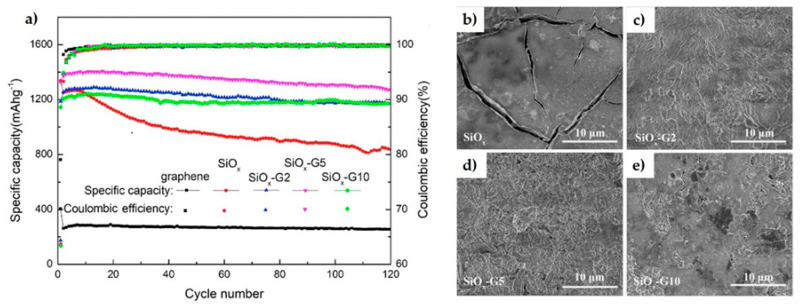
(**a**) Coulombic efficiency and specific capacity (at 100 mA·g^−1^) of SiOx and three SiOx/G composite electrodes (2 wt.%, 5 wt.%, 10 wt.%); (**b**–**e**) SEM micrographs of the surface of SiOx and the composite electrodes after 120 cycles. SiOx sample (**b**) shows large cracks in comparison to the smooth surface exhibited by the electrodes with graphene filler (**c**–**e**). Reprinted from [21], Copyright (2019), with permission from Elsevier.

The transport properties and cycling performance of SiOx [21] and SiOC [22] based electrodes have also been modified by mechanically exfoliated graphene and N-doped rGO aerogel, respectively. A SiO_x_ electrode with 5% graphene showed capacity retention of 95.8% with no apparent cracking after 120 cycles compared to pure SiO_x_ (Figure 2). On the other hand, the SiOC/N-rGO composite displayed initial Coulombic efficiency 18% higher than of SiOC alone, with up to 1000 cycles of stability. It seems that oxygen groups in rGO contributed to form the oxygen-rich SiO_4−x_C_x_ compound of higher reversible capacity.

##### TMO-Based Anodes

Choi et al. [23] fabricated a Co_3_O_4_@graphene hybrid by simultaneous reaction of metal species and carbon in a chemical vapor deposition (CVD) chamber followed by an oxidation step, obtaining 3 nm Co_3_O_4_ *np* encapsulated by the graphene layers. The anode was created by adding a binder and a conductive agent. The results showed an effective reversible volume change of the core-shell particles of 200% with the electrode volume change of 18%, achieving an initial charge capacity of 1131 mA·h·g^−1^ and excellent stability through cycling at low and high current densities (82% capacity retention after 2000 cycles at 2000 mA·g^−1^). In another work, Co_3_O_4_ and nanographitic flakes were mixed through a solvothermal method, and the corresponding electrode was fabricated by electrophoretic deposition (EPD) of this mixture to avoid the use of binders [24], thus achieving a charge capacity of 1197 mA·h·g^−1^ at 100 mA·g^−1^ with a capacity retention of 76% after 30 cycles. 

The intercalation of rGO layers with CuO nanospheres [25] and *np* [26] has also produced enhanced electrochemical properties of the composites over those of the plain metal oxide, with rGO playing a key role in protecting CuO from rapid degradation due to contact with Li^+^ ions, accommodating volume expansion, reducing diffusion paths and improving electron transport. The CuO *np*/rGO based anode showed a specific capacity of 623 mA·h·g^−1^ after 100 cycles with 95% Coulombic efficiency, whereas the porous CuO nanospheres/rGO based anode also presented high stability with capacity retention of 90.6% after 50 cycles. 

**Figure 3:**
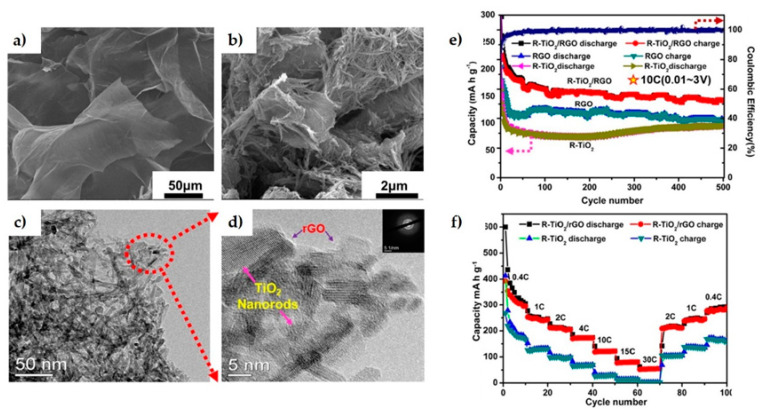
R-TiO_2_@rGO hybrid for LIB anode. (**a**) SEM image of GO, (**b**) SEM image of titanium hydroxide/GO after hydrothermal synthesis, (**c**,**d**) TEM images showing the morphology of R-TiO_2_@rGO hybrid; (**e**) cycling performance of R-TiO_2_@RGO and R-TiO_2_ at 10 C, (**f**) rate capacities. Adapted from [27], Copyright (2019), with permission from Elsevier.

An anode of TiO_2_ *nr* decorated rGO [27] as shown in Figure 3a–d, fabricated by hydrothermal synthesis and 500 °C annealing step, increased Li-ion diffusion coefficient in one order of magnitude compared to the TiO_2_-*nr* electrode, showing also 1.5 times higher capacity after 500 cycles (Figure 3e,f). GO has also been used to enhance the performance of a layered MoO_2_/Mo hybrid [28], accomplishing 90% higher discharge capacity with 85% initial Coulombic efficiency.

Other electrodes fabricated to take advantage of the mechanical stability and conductivity provided by graphene include, for example, a composite of Zn-Al hydroxide on graphene/polypyrrole nanosheets for Zn-Ni secondary batteries [29], showing 23% more capacity retention than the same material without graphene, and a hybrid of Co_2_(OH)_2_CO_3_ nanowires/graphene [30] that presented enhanced electronic conductivity and cycling stability.

#### 2.1.3. Ceramic Electrolytes

Solid-state batteries with Li or Na metal anodes have emerged as safer alternatives for higher energy density devices. In this sense, graphene has been proposed as an intermediate layer between Na_3_Zr_2_Si_2_PO_12_ (NASICON) and Na anode for reducing the interface resistance [31], and as reinforcing filler in Li_1+x_Al_x_Ti_2-x_(PO_4_)_3_ (LATP) solid electrolyte [32], increasing fracture toughness 120% respect to pure LATP for improved mechanical stability with envisioned reduction of Li dendrite penetration.

### 2.2. Supercapacitors

Supercapacitors stand out as complementary devices to batteries and fuel cells, being able to perform charge/discharge cycles in very short times and exhibiting higher specific power (2 to 15 kW·kg^−1^) and longer lifetime (5 to 10 years and up to 10^6^ charge/discharge cycles) [33]. Depending on the charge accumulation mechanism, they are classified as electric double-layer capacitors (EDLC), storing charge at the interface between electrode and electrolyte, pseudocapacitors, where charge transport is produced by fast reversible redox reactions, and hybrid or asymmetric capacitors, that combine both mechanisms on each electrode of the device. Preferred materials for EDLC are carbonaceous materials (activated carbon, graphite, CNT and, recently, graphene), which offer large surface area, ideal porosity for electrolyte diffusion and chemical stability, reaching a capacitance of 100 to 300 F·g^−1^; whereas, pseudocapacitors, analog to some battery electrodes, are developed from metal oxide compounds and often present higher energy densities [34]. GRM in the form of 1D fibers or 3D-structures are used to replace activated carbon, enhancing the device performance due to the high specific surface area, and superior electrical conductivity, though capacitance figures are moderate if sheet restacking and agglomeration are not controlled [35]. Following the same rationale described in the previous section, graphene and GO can act as support for anchoring metal oxide *np*, for improving their distribution, reducing volumetric changes and increasing electron transport. Presently, work on polymer/GRM composites is focused on pseudocapacitors and hybrid supercapacitors, achieving capacitance values in a range of 165 to 763 F⋅g^−1^ [36] and also on its combination with metal oxides and sulfides, which will be discussed below. 

Different metal oxides like TiO_2_, ZnO, Fe_2_O_3_, CuO, MnO_2_, RuO_2_, NiO, V_2_O_5_, or Co_3_O_4_, with varied nanostructures (nanoparticles, nanorods, nanofibers, platelets and flower-like) [37,38,39] are examples of materials that have been used for studying the pseudocapacitive capabilities of TMO. Blends with graphene or GO sheets are often carried out by hydrothermal synthesis, allowing TMO nanoparticles to clamp to graphene defective sites, functional groups and wrinkles. Among TMO, TiO_2_ is one of the most used compounds for electrochemical applications due to its low cost, availability and chemical stability, being also a target material for supercapacitors development. Fulari et al. [40] obtained TiO_2_/rGO powder composite with nanopetal-like microstructure by a coprecipitation method, and subsequent deposition on Cu and steel substrates as a way to reduce the device cost, reporting a better capacitive performance with the steel substrate (192 F·g^−1^ at 5mV·s^−1^). The presence of TiO_2_ *np* apparently worked synergistically with graphene, preventing sheets agglomeration and increasing the specific surface area of composites as well; for instance, a 3D rGO@TiO_2_ hybrid [41] showed a specific surface area of 171 m^2^·g^−1^, more than 3 times higher than that of pure 3D rGO, achieving a capacitance of 235 F·g^−1^ at 0.5 A·g^−1^. Yue et al. [42] fabricated a hybrid of TiO_2_ nanowires at rGO, which delivered capacitances of 572 and 330 F·g^−1^ at current densities of 1 and 10 A·g^−1^, respectively, due to the enhanced distribution of the high aspect ratio nanowires and better electrical conductivity by the presence of rGO. 

**Figure 4:**
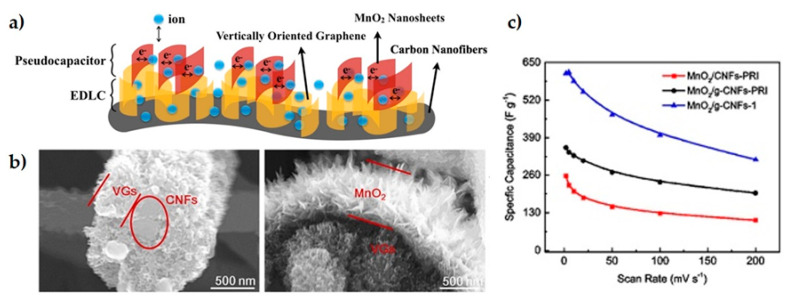
(**a**) Schematics of MnO_2_@G-CNF hybrid supercapacitor architecture, (**b**) SEM micrographs of the material. The electrostatic double-layer is composed of carbon nanofiber substrate and vertically oriented graphene sheets (VG). The pseudocapacitor consists of the MnO nanosheets grown on VG. (**c**) Performance of samples with different treatments and MnO_2_ contents. Reprinted from [43], Copyright (2018), with permission from Elsevier.

Qi et al. [43] fabricated a hybrid supercapacitor of electrospinning carbon fibers coated by vertically aligned graphene and MnO_2_ sheets. As can be observed in Figure 4, the exposed graphene edges seemed beneficial for growing thin MnO_2_ nanosheets, which improved the electrolyte wettability, enhanced electrical conductivity and reactivity, reporting a top capacitance of 612 F·g^−1^ at 2 mV·s^−1^ and capacity retention close to 100% after 5000 cycles. Another hybrid supercapacitor based on a single metal oxide/graphene composite was developed by Ding et al. [44] using Fe_2_O_3_ and graphene ribbons made from unzipped CNT (G/CNT) as the negative electrode, and MnO_2_/G/CNT as the positive electrode. The specific capacitance of each electrode was 258 F·g^−1^ for the negative one and 264 F·g^−1^ for the positive, at a current density of 1 A·g^−1^. Both showed higher capacitance than the pure TMO compounds or their composites with CNTs, capacitance retention of more than 80% after 5000 cycles and high energy density (43.2 W·h·kg^−1^). Other electrodes with excellent capacitive performance were fabricated from CuO/GO and ZnO/GO composites, and tested with different electrolytes (KCl, Na_2_SO_4_ and H_2_SO_4_) [45], showing superior values for the tests with KCl at 1 A·g^−1^ (800 Fg^−1^ for CuO/GO electrode and 450 F·g^−1^ for ZnO/GO).

The use of binary and ternary compounds augments pseudocapacitance by increasing oxidation states and faradaic reactions. Some binary TMO of interest includes NiCO_2_O_4_, ZnCo_2_O_4_, CoMoO_4_, NiMoO_4_ and NiWO_4_ [46], also mixed with graphene derivatives. For instance, NiCo_2_O_4_ *np*/carbon fiber composite, obtained from hydrothermal synthesis with annealed cotton fiber, showed capacitance of 1029 F·g^−1^ at 1 A·g^−1^, an improvement of 16% with respect to pure NiCo_2_O_4_. More recently, a layered structure of Co_3_O_4_ and NiO *np* deposited on CNT and subsequently coated with graphene [47] combined high electrical conductivity of the carbonaceous materials with pseudocapacitive properties of metal oxide compounds, reporting 22 times higher energy density and power density than pure CNT supercapacitor. The CoMo_2_S_4_ with S doped rGO electrode developed by Ma et al. [48] demonstrated a uniform dispersion of the sulfide particles, thus creating more channels for ion transfer and reducing structural damage. Proved with KOH electrolyte, they reported 1288 F·g^−1^ with a maximum energy density of 38.7 W·h·kg^−1^ and retention of 91.8% after 2000 cycles. Another composite based on NiCoS/graphene [49] demonstrated better electrical conductivity and numerous active sites for reversible redox reactions, obtaining 1186 F·g^−1^ of capacity at 1 A·g^−1^. A hybrid device formed by ternary metal sulfide and rGO, Mn-Ni-CoS/rGO, as a pseudocapacitive electrode and rGO on nickel foam as EDLC [50] showed capacitance of 1136.6 F·g^−1^ at 1 A·g^−1^ and retention of 98.8% after 5000 cycles at 2 A·g^−1^. This behavior was attributed to several causes, such as an enhanced contact with the KOH electrolyte, a higher oxidation state, and redox reactions, all combined with higher mechanical stability.

Further strategies include supercapacitors based on MXene (Ti_3_AlC_2_)/graphene composites, referring to top capacity of 599 F·g^−1^ at 1 A·g^−1^, 220% higher than the pure MXene [51], and composites of metal dichalcogenides [52].

### 2.3. Solar Cells

Compared to first-and second-generation photovoltaic cells, the emerging technologies in the field not only pursue increasing power conversion efficiency (PCE), but also look for the use of alternative materials and synthesis routes to reduce the production cost, ensure stability, scalability and minimize environmental impact. Different types of cells have been developed following this approach by introducing organic-inorganic hybrid materials and the assembly of multiple layers, favoring carrier transport mechanisms. The most promising structures are the dye-sensitized solar cells (DSSC), organic solar cells (OSC), perovskite solar cells (PSC) and quantum dot solar cells, with reported maximum PCE of 13%, 18.2%, 25.5% and 18.1% respectively, according to 2020 National Renewable Energy Laboratory report on the best research-cell conversion efficiencies [53]. During the past decade, graphene and GO were also considered as potential materials to be incorporated into solar cells due to their outstanding optical and electrical properties, showing high transparency (>98% for graphene monolayer) [54], ambipolar electric field effect and fast carrier mobility (200,000 cm^2^·V^−1^·s^−1^ for electrons in the suspended single-layer) [55,56]. These properties combined with the high specific surface area have been demonstrated to be effective for improving the performance of different cell components, from transparent electrodes to transport layers, providing at the same time better mechanical flexibility. rGO is currently the most used material in the processing of graphene composites for solar cells, with the potential for reducing production cost and to facilitate the synthesis of thin layers. The facile functionalization of rGO allows anchoring of p-type and n-type metal oxides, improving the efficiency of the electrodes in collecting and extracting charge carriers [57]. It has been observed that it increases electron–hole recombination resistance and reduces charge transfer resistance [58,59] compared to the material without graphene. Table 1 summarizes the photovoltaic performance achieved by some graphene composites in recent years.

#### 2.3.1. Dye-Sensitized Solar Cells 

Graphene and GO are added to metal oxides in the photo-anode, processing the composite mainly by hydrothermal synthesis, and applying it on top of a fluorine-doped tin oxide substrate. The composite is subsequently impregnated with a dye. As detailed in previous sections, graphene maintains metal oxide particles well dispersed and reduces agglomeration. The amount used is frequently below 1 wt.% as higher contents were detrimental due to void formation and lack of compactness. Graphene’s high specific surface area permits the enhancement of dye absorption, in particular for rGO, which also has the benefit of the facile functionalization that provides more anchoring sites for catalytic reactions. 

**Figure 5:**
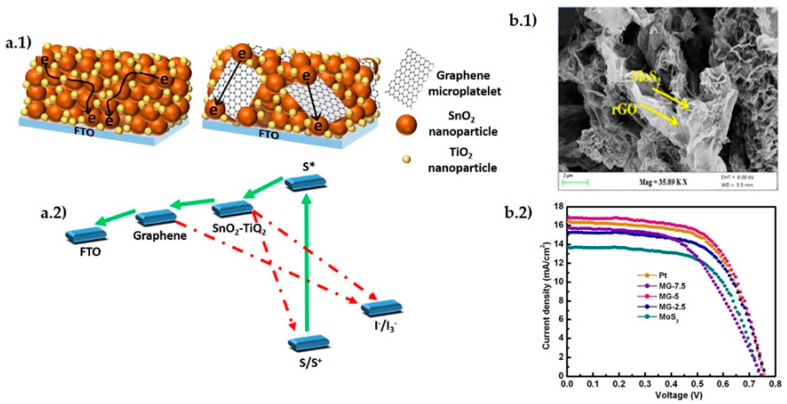
(**a.1**) Schematics of faster charge transport via graphene microplatelets in SnO_2^−^_TiO_2_/graphene anode; (**a.2**) diagram of an electronic band in DSSC with SnO_2^−^_TiO_2_/graphene anode. Reprinted from [62], Copyright (2020) with permission from Elsevier; (**b.1**) SEM image of MoS_2_/rGO counter electrode; and (**b.2**) J–V curves of DSSC using Pt counter electrode compared to those based on MoS_2_/rGO composites and pure MoS_2_, under simulated illumination (AM 1.5G, 100 mWcm^−2^). Reprinted from [59], copyright (2019), with permission from Elsevier.

TiO_2_, is again one of the most used materials, due to its facile production, chemical stability and environmentally safe character. It has been observed that GO creates strong chemical interaction with TiO_2_ particles, producing a uniform distribution that leads to a larger area for absorbing more dye molecules [60]. The TiO_2_/rGO sensitization with two different dyes (N719 and N3) has also proven to be effective for increasing cell PCE by 2% (or 38% of increment ratio) with respect to the use of a single dye. According to Kumar et al. [61], the augment of anchoring sites at carboxyl moiety and Π conjugation systems affect positively electron-hole recombination resistance and charge transport. A SnO_2_–TiO_2_/GNP hybrid anode Figure 5(a.1,a.2) [62] produced a PCE improvement from 2.91% for the material without graphene to 3.37% in the composite, exhibiting good stability after 200 h of continuous irradiation. Mohamed et al. [63] processed a composite of ZnO *nr*/GO-CNT, in which the GO-CNT hybrid reduced the size of ZnO crystallites, increasing the surface area by two orders of magnitude. It also had an effect on the redox performance of triiodide/iodide (I_3_^−^/I^−^) electrolyte by the formation of p–n heterojunction at ZnO (n-type)/CNT-GO (p-type) interface, efficient for electron-hole separation.

Graphene/metal oxide and graphene/metal dichalcogenide composites proved as valid alternatives to substitute Pt for the counter electrode (CE) in DSSC because of the enhanced electrocatalytic activity and reduced charge transfer resistance. For instance, a Co_3_O_4_/N-doped rGO composite [64] showed 42% reduced charge transfer resistance and DSSC achieved a 12% increase of the current density and 0.61% (7.8% increment ratio) higher PCE than with the Pt-based electrode. A CoSe/rGO counter electrode [65] showed improved catalytic performance increasing cell PCE by 0.24% with respect to a cell with Pt electrode and 0.54% with respect to the cell with a CoSe_2_ electrode. In a similar way, high electrocatalytic activity for I_3_^−^ reduction, 34% reduced charge transfer resistance and improved mechanical strength compared to Pt was measured for the MoSe_2_/rGO counter electrode, Figure 5(b.1,b.2) [59]. Singh et al. [66] presented an extended review on the capabilities of metal dichalcogenides and their composites for replacing Pt in DSSC counter electrode, with a comprehensive comparative table including materials reported up to 2017.

#### 2.3.2. Perovskite Solar Cells

PSC resembles the configuration of a DSSC (basically photoanode with a semiconductor on substrate/dye sensitizer/electrolyte/counter electrode), in which the perovskite layer occupies the place of the sensitizer. High-efficiency PSC (PCE 18%) was reported in 2016 for a cell with a TiO_2_/graphene composite electrode combined with a GO interlayer [67]. Graphene composites can be found as part of the conductive electrodes or as a carrier transporting material as in hole transport layer (HTL) [68] or as an electron transport layer (ETL), normally in composites with binary and ternary metal oxides enhancing the electronic transfer rate [69] and also reducing carrier recombination rate. More recently, graphene sheets have also been introduced within the active material layer to improve transport properties and chemical stability [70,71].

**Figure 6:**
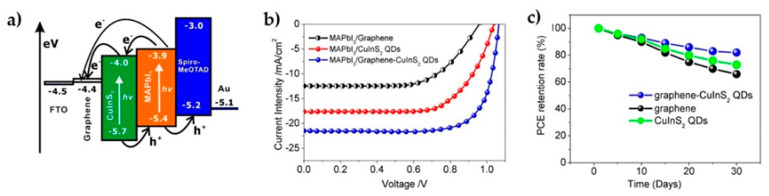
MAPbI_3_/CuInS_2_ QDs@Graphene PSC. (**a**) Diagram showing charge transfer energy levels; (**b**) J-V curve of PSC with different active layer compositions; and (**c**) performance of solar cell exposed to air. Reprinted from [71], Copyright (2020), with permission from Elsevier.

Heo et al. [72] reported graphene doped with AuCl_3_ as a top layer on polyethylene terephthalate (PET) substrate, thus improving carrier mobility by two orders of magnitude with respect to indium tin oxide showing at the same time enhanced flexibility with a PCE of 17.1%. Liu et al. [58] fabricated ETL from SnO_2_/rGO composite on AlZn oxide (AZO) substrate, reporting higher stability and lower roughness than pure SnO_2_ on AZO. The electron mobility exhibited five times higher value while PCE incremented from 13.9 to 16.9%. Another design proposed for ETL consisted of a ZnO *nr*/N-doped graphene composite [73]. The flower petal microstructure allowed higher loading of source solutions for the growth of a more uniform perovskite film, leading to improved light absorption and enhanced electron transport by the N-doped graphene, reaching PCE of 16.82%, about 4% (30% of increment ratio) higher than with pure ZnO *nr*. Excellent photovoltaic performance was achieved by Mahmoudi et al. [70] by mixing MAPBI_3−x_Cl_x_ (mixed halide perovskite) with rGO sheets decorated with Ag nanoparticles, which acted as a p-type semiconductor with improved carrier mobility (3 × 10^5^ cm^2^⋅V^−1^⋅s^−1^). The cell also exhibited long stability at ambient conditions for more than 300 days and important resistance to perovskite degradation when exposed to continuous illumination (93% of initial PCE compared to 39% of pure perovskite-based cell). Similarly, Gao et al. developed another composite active layer with outstanding performance [71] where effective charge dissociation at the interface of rGO-CuInS_2_ quantum dots and MAPBI_3_, produced a PCE of 17.1% Figure 6.

### 2.4. Fuel Cells

Compared to other energy storage devices fuel cells are highly efficient, producing electrical energy from chemical reactions when fuel and an oxidant are continuously supplied to the electrodes. The fuel reacts at the anode and the positive ions are transferred through an electrolyte membrane to recombine with the oxidant at the cathode. Different combinations of fuels and oxidants give the cell flexibility to operate at different temperatures, having the attractiveness of using sustainable fuels and showing highly stable electrodes. The most promising cells, named according to the type of electrolyte, are the proton exchange membrane fuel cells (PEMFC), solid oxide fuel cells (SOFC), alkaline fuel cells (AFC), microbial fuel cells (MFC), phosphoric acid fuel cells (PAFC) and direct alcohol fuel cells (DAFC). The two main challenges of this technology are related to the elevated price of the catalyst material and the control of ORR kinetics, which have motivated the research on graphene hybrids and nanocomposites for reduction of production cost and to improve the performance. An extended review on the use of GRM in the different components of fuel cells has been recently reported by Su and Hu [74].

**Table 1:** Ceramic/GRM composites used in solar cell components. The type of GRM is indicated first in the composite name.

Cell Type	Cell Component	Composite	Jsc (mA⋅cm^−2^)	Voc (V)	FF (Ratio or %)	PCE (%)	Ref.
DSSC	Photoanode	GO/Nb_2_O_5_	0.363	0.196	0.42	0.11	[75]
DSSC	Photoanode	GO-CNTs/ZnO *nr*	17.6	0.63	0.35	7.73	[63]
DSSC	Photoanode	rGO/TiO_2_	16.27	0.59	71.85	6.90	[61]
DSSC	Photoanode	rGO/TiO_2_	15.06	0.715	0.68	7.58	[60]
DSSC	Photoanode	GNP/SnO_2_-TiO_2_	9.03	0.65	0.58	3.37	[62]
DSSC	Photoanode	G/ZnAl-MMO	3.62	0.36	0.39	0.51	[76]
DSSC	Photoanode	GO/ZnAl-MMO	4.46	0.37	0.34	0.55	[77]
DSSC	CE	rGO/CoS_2_	16.35	0.702	0.67	7.69	[78]
DSSC	CE	G/NiSe	16.32	0.75	0.61	7.47	[79]
DSSC	CE	G/NiCo_2_S_4_	15.62	0.72	70.5	7.98	[80]
DSSC	CE	GO/CoFeS_2_	15.85	0.78	0.71	8.82	[81]
DSSC	CE	rGO/MoSe_2_	17.75	0.76	0.616	7.83	[59]
DSSC	CE	rGO/CoSe_2_	12.24	0.792	0.72	7.01	[65]
DSSC	CE	*nr* GO-MWCNT/Co_3_O_4_	17.09	0.75	65.7	8.42	[64]
PDSSC	Photoanode	rGO-Ag *np*/TiO_2_	14.08	0.73	66.35	6.87	[82]
QDSSC	Photoanode	GQD/C-ZnO *nr*	1.84	360	45.28	0.293	[83]
PSC	HTL	rGO/CZTS_0.5_Se_0.5_	17.43	0.917	63.07	10.18	[68]
PSC	ETL	rGO/SnO_2_/AZO	22.57	-	73	16.87	[58]
PSC	ETL	N-G/ZnO *nr*	21.98	1.02	75	16.82	[73]
PSC	Composite active layer	rGO-CuInS_2_ QD/MAPbI_3_	21.4	1.05	0.76	17.1	[71]
PSC	Composite active layer	rGO-Ag/MAPBI_3-x_Cl_x_	23.5	0.929	73.75	16.1	[70]

#### 2.4.1. Proton Exchange Membrane Fuel Cells 

**Figure 7:**
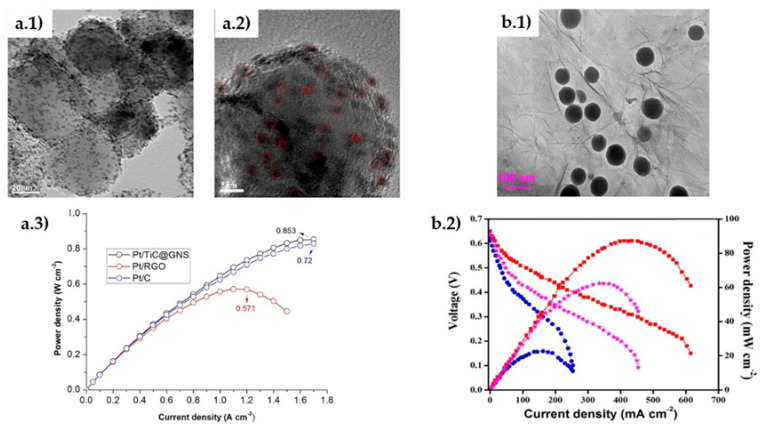
(**a**) Pt/TiC@GNS hybrid. (**a.1**,**a.2**) HRTEM images showing TiC core covered by graphene shell and Pt nanoparticles; (**a.3**) power densities obtained with Pt/TiC@GNS, Pt/rGO and Pt/C electrodes. Reprinted from [84], Copyright (2017), with permission from Elsevier. (**b.1**) TEM image of rGO@SiO_2_ hybrid, (**b.2**) DMFC performance using CS/SiO_2_@rGO membrane at 80 °C. Reprinted from [89], Copyright (2019), with permission of Elsevier.

Graphene agglomeration can be an important drawback as restacking, folding and bending can mask the effect of catalyst particles, impeding composite electrodes to achieve their maximum efficiency. The TiC@graphene core-shell structures [84] showed in Figure 7(a.1,a.2) demonstrated effectiveness in solving this problem and provided stability to the Pt *np* during the reaction. Using Pt loadings similar to the commercial Pt/C material, a maximum power density 18% higher than the Pt/C electrode (see Figure 7(a.3)) and an 8% higher diffusion limiting current were reported, demonstrating better cost-efficiency. It also improved mass transfer, compared to a layer by layer rGO electrode, augmenting Pt dispersion and increasing oxidation resistance as well. Regarding the electrolyte membrane, Simari et al. [85] synthesized a composite of TiO_2_@GO with sulfonated polysulfone as an alternative to the most common Nafion membrane, which may dehydrate easily reducing proton conductivity. The composite showed 22% higher water uptake and 5% higher ion exchange capacity, with the TiO_2_@GO hybrid limiting the degradation of sulfonic acid groups. For the sample with 3 wt.% hybrid filler, the proton conductivity was comparable to that of Nafion, being 110% higher at 20% relative humidity condition.

#### 2.4.2. Direct Alcohol Fuel Cells 

Rahmani et al. [86] fabricated a carbon–ceramic (CCE) substrate based on methyltrimethoxysilane and graphite that was subsequently coated with N-doped GO, and electrodeposited with NiCO alloy *np* on top. The CCE/N-GO/NiCo electrode displayed 87% and 47% higher current densities than the CCE/NiCo electrode during ethanol and methanol oxidation, respectively, due to the more accessible surface and active sites created by N-GO addition, with strong interaction with metal *np*. In the same way, CCE/rGO/PtNi and CCE/rGO/PtCu electrodes fabricated by a similar route showed improved performance for ethanol electro-oxidation with 70% and 17% higher forward anodic peak current densities, respectively, than CCE/rGO/Pt electrode [87]. AlN/graphite carbon core-shell electrode for methanol oxidation was prepared as a low-cost alternative to replace Pt-based cathode [88]. The material showed good tolerance to methanol and 8% higher ORR potential, compared to pure AlN. This electrode combined enhanced durability and protection from catalyst poisoning with high electrical conductivity. Ranjani et al. [89] improved the performance of the chitosan (CS) membrane by adding SiO_2_@sGO hybrid nanofillers. Sulfonated GO (sGO) created stable bonding with SiO_2_ *np* by hydrogen bridges through–SO_3_H functional group (Figure 7b), and water adsorption improved more than 30% compared to CS alone. In addition, the nanocomposite membrane showed 160% higher ionic conductivity at 80 °C and better methanol permeability.

#### 2.4.3. Microbial Fuel Cells 

A Portland cement/graphite flake composite was used as a substrate for the cathode in MFC [90]. The cell performance was tested using Nafion membrane and a carbon fiber anode while food residues were used to fill the anodic chamber. The results showed an increment of six orders or magnitude in electrical conductivity, obtaining maximum power density of 28 mV⋅cm^−2^ and output voltages in the range 750–650 mV during 10 days. Li et al. [91] prepared a composite anode based on Ti_4_O_7_/rGO/polyaniline for improving bacterial loading and power density. The rGO additions increased surface area in one order of magnitude with respect to a Ti_4_O_7_ electrode and showed a 10% higher strength. In combination with polyaniline, good charge transfer and increased electric conductivity were observed, delivering a maximum power density of 2073 mW⋅m^−2^ that was five times that of TS. In accordance with Ahilan et al. [92], polymeric membranes for MFC present mechanical stability issues in large devices due to high hydrostatic pressures, therefore, it is a real challenge to find appropriate materials for scaling up. The authors fabricated SiOC/rGO and SiOC/multiwall carbon nanotubes (MWCNT) composite membranes obtaining one order of magnitude higher flexural strengths compared to the pure polymer derived ceramics (PDC) membrane. Even more, the 0.5 wt.% rGO composite showed 32% higher strength than the composite with the same content of MWCNTs. The rGO containing composite also showed increased hydrophilicity and the corresponding cell produced operating voltages of 245 mV, 15% and 35% higher than with SiOC and Nafion membranes, respectively. 

#### 2.4.4. Solid Oxide Fuel Cells 

Cubic yttria-stabilized zirconia (YSZ) has been widely used as a solid electrolyte due to its high stability in reducing and oxidizing atmospheres. Accordingly, large work has been focused on finding materials with good affinity to c-YSZ for electrodes or interconnects between adjacent cells [93]. Marinha and Belmonte [94] studied the effect of the addition of GNP (7–14 vol.%) on the electrical conductivity of c-YSZ and its stability under oxygen partial pressures (P_O2_) in the range 0.21–10^−20^ atm, finding that electrical conductivity increased in four orders of magnitude after percolation threshold, with a maximum of 8 S⋅m^−1^ at 300 °C. The samples showed stability in inert and reducing atmospheres at temperatures as high as 600 °C and 800 °C, respectively. Following this line, Kurapova et al. [95] fabricated c-YSZ/rGO composites showing important grain size refinement and electrical conductivity of 10 S⋅m^−1^ at 1000 K. Gómez-Gómez et al. [96] suggested that these composites could be used as suitable interconnects by proving important increases of the thermal and mechanical properties by the GNP addition, in particular, for the 20 vol.% GNP composite six times higher thermal conductivity (17 W·m^−1^·K^−1^) than monolithic materials, and 78% increment in fracture toughness with rising R-curve behavior was demonstrated for the 11 vol.% GNP.

On the other hand, Ahmad et al. [97] fabricated Al-Ni-Zn oxide/graphene composite anode with the aim of reducing the high operating temperature (800–1000 °C) in SOFC, reporting an optimized content for the 1.3 wt.% graphene composite that showed a maximum open-circuit voltage of 0.95 V with a power density of 375 m·W·cm^2^ at 600 °C.

## 3. Piezo and Thermoelectric Ceramic/Graphene Composites for Energy Harvesting

### 3.1. Piezoelectric Energy Harvesting

The limitation of resources and the interest in producing miniature and portable electronic devices have motivated the research on thin films, bulks and scaffolds that work as micro-and nano-generators by the action of the piezoelectric effect. In such materials, the temporary formation of dipoles by the effect of mechanical strain produces an electric potential. Numerous reviews on the composition, working principle and applications of piezoelectric ceramics, polymers and composites have been published in the past decade [98,99,100]. Lead zirconate titanate (PZT) has become one of the most-used polycrystalline materials exhibiting high power output and high piezoelectric coefficient, followed by lead-free alternatives as barium titanate (BT) or zinc oxide. However, polycrystalline ceramic materials or single crystals have an inherent brittleness and shaping difficulty, therefore, piezoelectric polymers and composites have emerged as interesting alternatives, as they offer high flexibility and good piezoelectric voltage constants (g_33_ and g_31_) [100]. The use of graphene in piezoelectric generators was motivated at first by the development of flexible and transparent electronics [101], but now it finds application in a varied range or structures with impact in medicine, civil engineering, sensing or automotive industry, as low graphene contents can enhance piezoelectric properties. The most used graphene source is in the form of rGO, mixed with the ceramic piezoelectric matrix or alternatively, hybridized with piezoelectric particles that are incorporated into the polymer matrix.

#### 3.1.1. Ceramic/Graphene Composites 

Alam et al. [102] studied the dielectric constant (ε_r_,Κ) of PZT/GO composites, in the frequency range of 100 kHz to 1 MHz, finding that, at low frequency, Κ increased in one order of magnitude compared to PZT (360 for PZT, 1443 for the 1 wt.% GO sample) due to space-charge polarization at grain boundary interfaces. In another study, a cement-based piezoelectric composite was fabricated for application in structural monitoring [103]. Lead niobate zirconate titanate (PNZT) was mixed at 50 vol.% with ordinary cement, adding polyvinylidene fluoride (PVDF) and GO to improve the polarization and create a continuous electric flux. The results showed increasing dielectric constant with the increment of GO filler but the undesirable augment of dielectric losses for contents above percolation threshold restricted its use to 2 vol.% GO.

#### 3.1.2. Polymer/Ceramic/Graphene Composites Based Composites

PVDF possesses high chemical stability and good piezoelectric and ferroelectric properties as well as biocompatibility, being one of the most studied polymer materials with applications in medicine, optics and aerospace. PVDF piezoelectric constant is two orders of magnitude lower than that of PZT and the common route to enhance its properties is the addition of piezo-ceramic particles (piezoelectric coefficient of 22.93 pC·N^−1^, 184 mV under a load of 2.125 N was measured for PVDF nanocomposite with 37 vol.% PVDF-PZT electrospinned fibers) [104]. Improved piezoelectric and mechanical behavior can be achieved when adding GRM to these composites, as graphene promotes PVDF β-phase crystallization, which exhibits key dipole orientation and better piezoelectric properties [105]. For instance, Tang and coworkers [106] fabricated PVDF and PZT with the addition of graphene sheets. This resulted in an increment of the electric field on the surface of the PZT particles, with a 21.6% higher piezoelectric constant (d_33_) for the optimum graphene content of 0.6 wt.%. Above this content, a rapid increment of the dielectric loss was observed that impeded polarization. When the polivinylidene fluoride-trifluoroethylene (PVDF-TrFE) polymer blend was mixed with a ZnO@GO hybrid [107], an enhancement of β-phase formation (of 233%) was observed, as well as a better ZnO distribution; these factors produced output voltages under finger tapping 170% and 30% higher than the matrix PVDF-TrFE and PVDF-TrFE/ZnO composite, respectively. Kim et al. [108] also processed PVDF-TrFe composite film with enhanced piezoelectric properties by simultaneously adding Pb(Mg_1/3_Nb_2/3_)O_3_-PbTiO_3_ (PMN_PT) and rGO. These phases augmented the dielectric constant above twelve times compared to the pure polymer film, due to the increment in interfacial polarization between the different phases. The measured piezoelectric constant for the composite with optimized graphene content was 22 pC·N^−1^ and the peak voltage was 8.4 V under an external load of 350 N applied at 4 Hz. The hydrothermal method was chosen to fabricate the Bi_2_Al_4_O_4_ *nr*@rGO hybrid filler for lead-free PVDF-based nanogenerator [109], obtaining an increment of 76% in PVDF β-phase nucleation. The combined piezoelectric properties of the ceramic *nr* and the polymer with rGO produced better order of local dipoles with the interaction of Π-electron in rGO and PVDF CH_2_^−^ dipoles, improving the conducting path and giving a remnant polarization one order of magnitude higher than pure PVDF and four times higher output voltage with a power density of 0.457 µW·cm^−2^.

Wearable electronic applications drive the interest to manufacture smart flexible devices attached to clothes and the skin, or implanted into the human body for detecting chemicals and monitoring vital signals [110]. Graphene has risen as one of the most promising materials for these kinds of applications because of its light weight, flexibility, biocompatibility, optical transparency, piezo-and thermo-resistive response, and electrical sensitivity. Graphene-based flexible electronic devices are commonly developed using organics as supporting material. The combination of ceramics and graphene has been reported by Choudhry et al. [111], who mixed distinct piezoelectric ceramics (PZT, BaTiO_3_ and ZnO) with GNP into a silicone elastomer matrix to produce flexible nanocomposite-based piezoelectric nanogenerators. The PZT-based material containing 0.5 wt.% of GNP led to the best piezoelectric performance as graphene promoted the formation of nanoelectrical bridges between piezoparticles. Furthermore, as a proof of concept, the authors developed a fully functioning shoe-insole nanogenerator under real-time human walking. 

**Figure 8:**
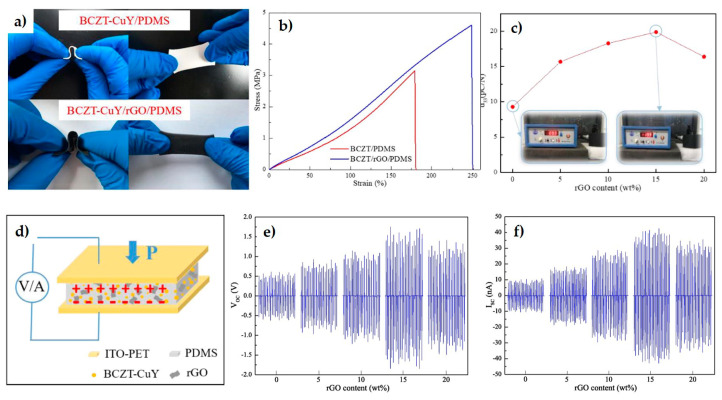
Strength and piezoelectric properties of PDMS/BCZT/rGO: (**a**) Stretching of PDMS/BCZT and PDMS/BCZT/rGO composites; (**b**) stress- strain curve; (**c**) piezoelectric constant at different rGO contents; (**d**) schematic diagram of dipole formation; (**e**) open-circuit voltage and (**f**) short circuit current under finger tapping. Reprinted from [112], Copyright (2018), with permission from Elsevier.

In the search for designing more efficient nanogenerators, and based on interesting results on (Ba, Ca)(Zr, Ti)O_3_ (BCZT) to replace toxic PZT materials, rGO was added to a mixture of (Ba_0.85_Ca_0.15_)(Ti_0.9_Zr_0.1_)O_3_–Cu–Y_2_O_3_ (BCZT-CuY) and polydimethylsiloxane (PDMS) [112]. It was found that rGO not only improved both the elastic modulus (48%) and elongation at break (38%), but it also contributed to the homogeneous distribution of the piezo-ceramic particles. As it is depicted in Figure 8, the best properties were reported for rGO contents near the percolation threshold (15 wt.%), increasing 114% the piezoelectric constant (d_33_ = 19.9 pC·N^−1^) compared to the BCZT-CuY film. At this content, a finger tapping of 15–18 N produced the maximum open-circuit voltage (Voc) of 1.36 V and a short circuit current of 35 nA. On the other hand, a composite scaffold of poly-L-lactic acid (PLLA) with 10 wt.% BaTiO_3_ and 0.4 wt.% GO was formed by SLS (selective laser sintering) for bone repair via electrical stimulation [113]. The ordering of BT domains with the external electric field was favored by GO, which also improved the local electric field. The PLLA/BT/GO sample output voltage and short circuit current augmented 84% and 96%, respectively, compared to plain scaffolds; displaying at the same time improved compressive strength (25%) and modulus (63%), as well as enhanced cell activity.

### 3.2. Thermoelectric and Pyroelectric Materials

#### 3.2.1. Thermoelectric Energy Harvesting

In thermoelectric materials, an electric potential is generated by the existence of a thermal gradient, which is an attractive choice for complementary energy storage modules that can improve device efficiency by utilization of natural heat sources and waste heat. The performance of thermoelectric materials is described by the figure of merit (ZT), relating the electrical conductivity (σ_e_), the temperature gradient (ΔT), the thermal conductivity (κ) and the Seebeck coefficient (S), according to the expression ZT = S^2^⋅σ_e_⋅ΔT/κ. 

GRM has called attention to the development of thermoelectric generators because of their doping possibilities. Seebeck coefficient of 90 µV⋅K^−1^ was reported for multilayer graphene, in the range of 50 to 1000 layers [114]. Considerable work has been devoted to SiTiO_3_ (STO) based thermo-generators due to its high-temperature stability, its non-toxicity, and thermal properties tailoring by multiple doping. Srivastava et al. [115] studied the thermoelectric behavior of La, Nb-doped SrTiO_3_ with different additions of GNP and rGO, verifying the anisotropic electrical and thermal conductivity induced by the use of the spark plasma sintering (SPS) densification method.

The improvements in ZT were related to the increment in electrical conductivity and the trade-off with thermal conductivity. Maximum ZT of 0.25 at 1000 K was obtained for 1 wt.% GNP in the cross-plane direction (the lowest thermal conductivity condition). Higher rGO contents would be needed to match the electrical conductivity of GNP, but this would be detrimental to thermoelectric behavior as it reduces the n-type semiconductor nature of the matrix. Nb-doped SrTiO_3_ (STNO)/rGO composites with rGO content varying between 0.3 and 1 wt.% were developed by Wu et al. [116]. All samples showed a lower Seebeck coefficient than the matrix, but rGO promoted a reduction in thermal conductivity by separation of the Nb rich TiO_2_ phases, grain refinement and formation of thermal resistances at grain boundaries and rGO/matrix interfaces, hence the 0.6 wt.% rGO composite showed the highest ZT value of 0.22 at 800 K. Okhay et al. [117] observed the thermoelectric properties of STNO/ rGO composites varying the amount of Nb dopant. Both Sr deficiency and rGO contributed to decreasing thermal conductivity due to associated oxygen vacancies which generate phonon scattering. The maximum ZT value measured at 1100 K was 0.29 and electron mobility was improved three times compared to the plain ceramic. Rahman et al. [118] proposed that, in addition to doping, increasing the carrier mobility and electrical conductivity of STO controlled the formation of double Shottky barriers at grain boundaries, due to strontium and oxygen vacancies, which acted as scattering centers for electron mobility. The use of 0.7% rGO as a high conductivity phase at grain boundaries resulted in the reduction of Shottky barrier height, releasing the trapped electrons and enhancing mobility. The composite showed two times higher ZT at 750 °C than the pure STO. Some authors proposed an increase of the power factor in perovskites by using SPS as a way to enhance vacancies, who accordingly prepared un-doped STO/rGO composites by first modifying the STO surface with aminosilane groups and then mixing both STO and GO using the colloidal route. The hybrid was treated at 473 K in the SPS, achieving a ZT value of 0.09 at 760 K for just 0.64 vol.% of rGO due to the effectiveness of rGO dispersion. The increase of the STO oxygen vacancies was attributed to a minor reaction between rGO and STO, which favored the electrical conductivity and claimed as particularly effective when compared to long-time hydrogen reduction [119].

The thermoelectric characteristics of BaTiO_3_/GO composites (fabricated by conventional powders mixing and hot pressing (HP) densification at 1100 °C) were evaluated for different GO additions (0.6–4 wt.%) in the 300–600 K temperature range. These composites displayed n-type semiconducting for GO contents < 2 wt.%, with a maximum ZT value (0.008) at 600 K for the 1.7 wt.% GO material [120]. Composites of a Mn_0.7_Zn_0.3_Fe_2_O_4_ ferrite with graphene additions (1–3 wt.%) were prepared in this case by co-precipitation methods; graphene sheets were first dispersed in aqueous NaOH solution while metal chloride precursors were dropwise added, reacted at 100 °C, dried and finally treated in the SPS at 750 °C. The composites showed a maximum ZT value of 0.035 for the 2 wt.% composites at ~1000 K [121]. Thermoelectric characteristics were also reported in Si_3_N_4_/GRM composites by preparing different composites with individual GNP (17–21 vol.%) and rGO (4–7 vol.%) additions, using ultrasonic dispersion and different solvents for each material and SPS densification at 1625 °C. The thermopower effect was determined in the 100–300 K interval achieving a maximum ZT value of 0.09 at 300 K for the 7 vol.% rGO composite, which showed n-type semiconductor behavior, whereas the GNP composites were p-type semiconductors. The higher thermopower (0.38 μW⋅m^−1^⋅K^−2^) of the rGO composites compared to GNP ones was attributed to the occurrence of O/N clusters in the GO sheet affecting the density of states [122].

Ghosh et al. [123] tested thermoelectric properties of GNP/Portland cement composites, reporting a figure of merit ZT of 0.44 × 10^−3^ for the 15 wt.% composites, which was considered of interest for potential applications in energy harvesting and better indoor temperatures, considering the amount of free heat absorbed by building facades. Along this line, other authors used ZnO in combination with GNP to enhance the thermoelectric response of the cement composite reporting a notable increase of ZT to 0.01 for a 10 wt.% of inclusions, which further drives the application in energy harvesting of smart buildings [124].

#### 3.2.2. Pyroelectric Energy Harvesting

Thermal energy harvesting from diverse sources (solar, geothermal, industrial waste heat, friction and human body heat) using pyroelectric materials [125] is another field where ceramic/graphene composites can play an important role. Pyroelectric materials allow direct conversion of thermal energy to electrical energy, based on time-temperature changes, and additionally, since all pyroelectrics are also piezoelectrics, they enable the conversion of mechanical to electrical energy. Recently, research activities on improving thermal energy harvesting performance by surface modification of pyroelectric materials using carbon-based coatings have been identified [126,127,128,129], some of them attempting surface modification of PZT and BT by graphene-based coatings. Liu et al. [129] processed a thin, soft, piezoelectric composite of graphene, PZT and PDMS for a skin-integrated electronic device, capable of converting body mechanical activity into electricity. A device based on a (Bi_0.5_Na_0.5_)_(1−x)_Ba_x_TiO_3_ (x~0.06) ferroelectric with the addition of GO has also been proposed. The ferroelectric BNT-BT/GO layered hetero structure was fabricated using spin coating methods and Langmuir-Blodgett assembly. The nanogenerator device included a silica-coated Si wafer substrate with a Pt/Ti electrode over the substrate and deposited Au electrodes on the graphene layer. A thermal-to-electrical energy conversion as high as 1.361 J⋅cm^−3^ was reported, with temperatures varying between 30 and 120 °C, which was estimated as rather promising for waste heat recovery in civilian and industrial facilities [128]. The pyroelectric effect generally produces a higher thermal-to-power energy conversion efficiency than the thermoelectric one [130], which would require very large gradients to increase efficiency.

## 4. Sensors Based on Ceramic/Graphene Composites and Hybrids

Since its discovery, graphene has received full attention as suitable sensing material due to its remarkable electrochemical activity [131,132]. Graphene is capable of perceiving molecular scale perturbations [133] as a result of its 2D nature [134], and it also exhibits a wide electrochemical potential window, low charge-transfer resistance, low signal/noise ratio, and fast electron transfer rate [135,136]. All these characteristics identified graphene as a premium material for electrochemical sensing [134], often outperforming its counterparts, such as carbon (glassy carbon, pyrolytic graphite) and CNT, although frequently used jointly [137]. Actually, GO is the preferred material for sensing applications due to its lack of metallic contaminants, the number of oxygen groups that facilitate its functionalization with different compounds and nanoparticles, its potential of band gap tuning, and its lower production cost (i.e., from graphite sources) [135,138]. Several comprehensive reviews can be found on the detection capacities of graphene [131,133,139], including some very recent ones [134,140,141].

In particular, graphene has proved its potential as bio-sensor for detecting different body constants (temperature, blood pressure, pulse rate) [142], biomolecules and biomarkers [131,138], as gas and humidity sensors [139,143], pressure sensor [144], strain sensor [145], toxins sensor [146], for crack detection in structures [147], etc. Some other sensing applications include the safety check of food and beverages, and devices for smart agriculture [148,149]. Graphene-based devices built on soft substrates are also very attractive for flexible and wearable electronics [137,150], and human health monitoring [138,151]. 

The variables determining the sensor performance include sensitivity, linearity, resolution, selectivity, detection limit, stability, and recovery and response times. Although different variables can be used (e.g., optical, thermal, acoustic, electrical, magnetic, etc.) for recording the desired stimulus, the chosen signal is generally converted into an electrical signal using convenient interfaces and digital units for the full control and integration of the sensing device, and sometimes even incorporating portability or wearability benefits [137,142]. Electrochemical sensors can be potentiometric, voltametric, amperometric or by impedance spectroscopy, whereas photochemical biosensors are commonly based on fluorescence resonance, laser mass spectrometry and surface-enhanced Raman spectroscopy. Increasing the sensitivity of detection systems is a constant urge, for instance, electrochemical sensors are often required to detect harmful gases and pollutants in less than parts per million concentrations [141,152]. 

In most cases, GRM appears combined with other materials besides carbon-based ones, such as polymers, metallic nanoparticles, metal oxides and ceramics for increased performance (sensitivity, stability, selectivity, etc.) of the sensing device [136,153]. This section focuses on nanocomposites and hybrids of the last two systems, providing a summary of recent developments on this matter. Metal oxide (MO_x_)/GO nanomaterials are predominantly used as gas sensors for different harmful gases, since the hybridization of both types of materials generate beneficial synergic effects [153,154]. The remainder ceramic-graphene nanocomposites are mostly used as electrochemical sensors and biosensors. A brief description of the fabrication route for each sensor type is also provided in this study because it greatly determines the sensor performance [142,155].

### 4.1. MO_X_ Semiconductor–Graphene Type Sensors 

#### 4.1.1. SnO_2_/rGO Sensors 

**Figure 9:**
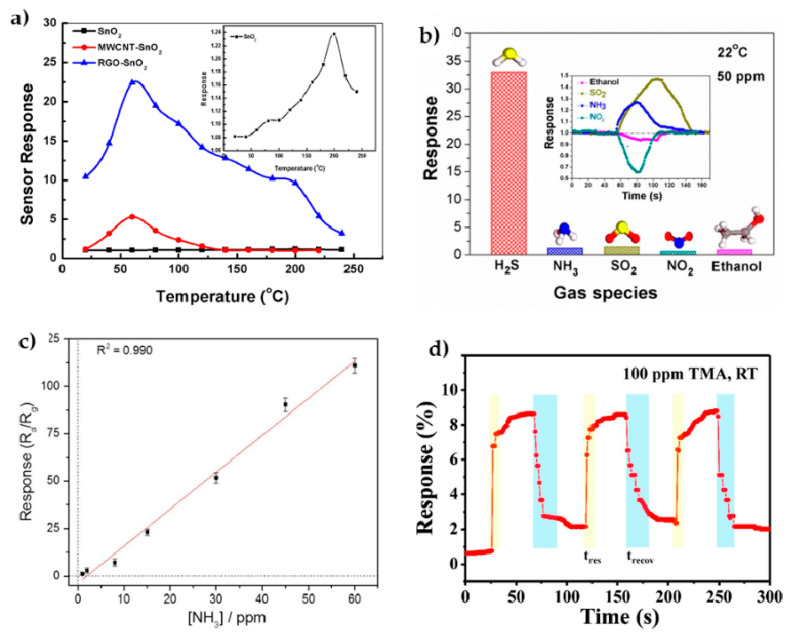
Examples of performance for different sensors: (**a**) Comparison of the sensing response for detection of SO_2_ gas of SnO_2_, SnO_2_/rGO and SnO_2_/MWCNT sensors as a function of temperature. Reprinted from reference [156], Copyright (2017), with permission from Elsevier; (**b**) Selectivity of the SnO_2_ quantum wire/rGO gas sensor for SH_2_ detection compared to other gases, reprinted with permission from [155], Copyright (2016) American Chemical Society; (**c**) linear response of In_2_O_3_ nanofibers/GO hybrid sensor to the NH3 concentration. Reprinted from reference [168], Copyright (2019), with permission from Elsevier; (**d**) time response/recovery curves of an In_2_O_3_/rGO composite sensor to successive cycles of 100 ppm TMA at RT. Reprinted from reference [170], Copyright (2019), with permission from Elsevier.

SnO_2_ is an n-type semiconductor material widely used in sensing applications because of its responsivity to many gas species, accordingly many works combine it with other materials, and particularly with GRM, for enhancement of its sensing properties. SnO_2_-rGO sensors have demonstrated improved performance with respect to the bare SnO_2_ sensor because they face problems such as poor selectivity and high-temperature requirement [156]. As an example of this, the temperature effect on the sensor response is compared in Figure 9a for SnO_2_, SnO_2_/CNT and SnO_2_/rGO, which evidences the effectivity of the last one for detecting SO_2_ gas. Thin films of these nanocomposites were prepared by spin coating from colloidal mixtures of GO and SnO_2_ *np* [156]; whereas, SnO_2_/rGO nanocomposites prepared by means of a microwave treatment showed a remarkable performance for NO_2_ detection in terms of sensitivity (1–5 ppm), time response and recovery time [157]. 

The use of SnO_2_/rGO hybrids assembled through the hydrothermal synthesis method as humidity sensors have also been reported, claiming a superior sensitivity to that of rGO bare sensors [158]. Nanocomposite sensors prepared by solvothermal methods have also been used for the detection of various volatile organic compounds such as formaldehyde [159] or ethanol [160], with enhanced sensing capabilities in both cases, results that were attributed to the improved specific surface area, the developed porous structure [159], and the microstructure of well-dispersed SnO_2_ hollow *np* decorating the rGO sheets [160].

The requirement of working temperatures above ambient temperature is a serious drawback for many of these sensors because limits their applicability. A sensor device based on a SnO_2_ quantum wire/rGO nanocomposite for detection of H_2_S has successfully addressed this problem. This sensor was processed via a one-step solvothermal route from Sn-precursors and aqueous GO solution reacting in an autoclave at 180 °C; the product consisted of SnO_2_ wires attached to the rGO surface (3.88 wt.% nominal SnO_2_/rGO ratio). The wires had reduced diameters by the GO attachment (in the range 3–6 nm), which induced quantum confinement and tunable band gaps. The sensor was finally fabricated by spin coating on an alumina substrate without further heating treatment, and showed a remarkable sensitivity and selectivity Figure 9b for H_2_S detection at room temperature (RT), also showing fast response and recovery times [155].

In most of the described SnO_2_ composite sensors, graphene contents (actually, rGO) are below 5 wt.%, where the graphene sheet serves as a unique platform for *np* attachment.

#### 4.1.2. WO_3_/rGO Sensors

**Figure 10:**
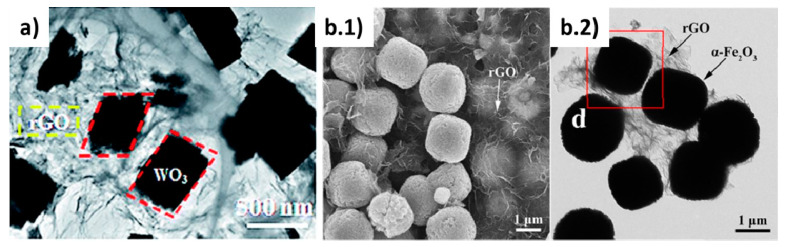
Example of microstructures of sensors prepared by hydrothermal synthesis: (**a**) transmission electron microscopy (TEM) image of WO_3_/rGO sensor reprinted from reference [163] published by the Royal Society of Chemistry; (**b.1**) FESEM and (**b.2**) TEM views of Fe_2_O_3_-1.0 wt.% rGO composites reprinted from reference [180], Copyright (2017), with permission from Elsevier.

Quite similar motivations exist for WO_3_/rGO composites intended for sensing applications. WO_3_ is a metal oxide n-type semiconductor that has mostly been used for detecting toxic gases, and its hybridization with rGO has been profited to cut some of its weakness (high RT resistance and poor selectivity) [161]. Composites formed of intercalated WO_3_ and rGO nanosheets (in proportions that ranged from 1.6 to 7.2 wt.% of rGO), prepared via ultrasonic stirring and hydrothermal assembling, showed improved performance for detection of H_2_S, being the 3.8 wt.% composites the most remarkable in terms of sensitivity and short response time [161]. In another example, composite fibers of WO_3_/rGO were prepared by electrospinning a mixture of the W precursor and the rGO solutions, and further calcination at 500 °C. This fiber-type composite sensor showed better sensing characteristics than WO_3_ for the detection of acetone [162]. Good sensing characteristics for NO_2_ detection were demonstrated by single-crystalline WO_3_/rGO porous nanocomposites (4.1–9.7 wt.% of rGO) prepared by the hydrothermal method [163] Figure 10a. The gas sensor was fabricated by coating a ceramic tube with the composite and corresponding wiring. By simple sonication and using spin coating methods, the fabricated WO_3_/rGO nanocomposite sensor (with 5 wt.% rGO) showed excellent sensing characteristics for NH_3_ detection as compared to the single WO_3_ sensor, attributed to the pore distribution achieved, the higher specific surface area and the occurrence of multiple p-n heterojunctions [164]. Nanocomposites of WO_3_/rGO obtained by hydrothermal synthesis and spin coating on interdigitated alumina substrates have also shown very good capabilities for the sensing of acetylene, particularly for the GO concentration of 1 wt.% [165] that showed a low detection limit of 1 ppm, stability and lower working temperature. By using a different method based on the liquid flame spraying of a solution of GO and a tungsten precursor, a composite consisting of WO_3_ *np* attached to the GO nanosheets was achieved showing an improved O_3_ detection for rGO contents in the range 1–3 wt.% [166].

#### 4.1.3. In_2_O_3_/rGO Sensors

Indium oxide is an n-type semiconductor with gas sensing characteristics as the previous metal oxides. Flowerlike In_2_O_3_/rGO nanocomposites with different rGO proportions (1–10 wt.%) were formed by hydrothermal synthesis by using hydrated indium nitrate as a precursor. The mixtures once treated at 300 °C were smeared onto a ceramic tube using water as a vehicle to form a thick coating. These sensors showed an excellent detection for NO_2_, with a detection limit as low as 1 ppb [167]. Recently, In_2_O_3_ nanofibers were processed by electrospinning from a precursor solution and subsequent thermal calcination around 500 °C, then the nanofibers were mixed with a solution GO and reduced to achieve a hybrid composite. An aqueous suspension of the hybrid was drop cast over the Au interdigitated electrode prepared by photolithography. The sensor showed a higher sensitivity for NH_3_ detection Figure 9c at RT than any of the materials independently. Higher selectivity for ammonia was proved as well [168]. In another report, In_2_O_3_ *np* prepared via hydrothermal synthesis were mixed with GO, and a thin film was prepared by drop coating, the hybrid materials were tested as humidity sensor having both fast response and recovery times [169].

In_2_O_3_/rGO composites have also been used for detecting trimethylamine (TMA), an amine that volatilizes from dead seafood. The composite was prepared by hydrothermal synthesis thus forming an intercalated self-assembled composite that after treating at 500 °C was directly tried as a sensor for TMA. Sensing reproducibility, selectivity and fast response were demonstrated in Figure 9d [170]. 

#### 4.1.4. ZnO/Graphene or GO Sensors 

Flower-like ZnO/GO hybrids were used as a gas sensor material for NO_2_ detection with a remarkable detection limit of 5 ppb. The nanocomposite was prepared using a solution-precipitation route from hydrated zinc acetate precursor and GO powders. The centrifuged composite powders after calcination (~300 °C) and freeze-drying were mixed with water and spread on a ceramic tube with attached Au electrodes, wires and a heating system to fabricate the sensing device. The optimum sensing response was achieved by the 1.7 wt.% rGO nanocomposite at the working temperature of 100 °C. The response was measured as the resistance ratio of the sensor under the test gas (50 ppb NO_2_) and the air atmosphere in static conditions [171].

A ZnO/graphene hybrid material connected to an array of micro-supercapacitors, and all integrated on a foldable paper substrate has been used as a UV sensor suitable for wearable electronics. ZnO nanoparticles acted as the photoluminescent material providing e-h pairs and graphene was the transporting material. Graphene was produced by CVD on Ni substrates and transferred to a gold patterned PET substrate. The ZnO *np* were prepared by co-precipitation methods and deposited on the graphene film by spin coating. The device was capable of detecting UV light autonomously during 1500 s [172]. 

An optical-based sensor for the detection of NH_3_ was prepared by coating a tapered interferometric optical microfiber with a ZnO/rGO nanocomposite. The composite was prepared by hydrothermal synthesis from a solution of hydrated zinc nitrate and GO, this composite mixture (once washed, dried and 160 °C treated) was drop cast (water vehicle) on the tapered microfiber section (vacuum dried at 45 °C). The detection principle of this sensor is based on the change of its optical properties, in particular of the refractive index when exposed to the analyzed gas, and therefore the corresponding interference spectra and wavelength shifts are recorded. Sensitivity to ammonia in the range of 4–140 ppm at RT, in addition to high selectivity, were verified [173].

Heterostructures formed by ZnO nanotubes and graphene have been tested as H_2_ sensors with an excellent response at detection levels in the range of 10–100 ppm. For the hybrid preparation, graphene was first formed by CVD over a Cu foil, transferred into a Si/SiO_2_ substrate, and sputtered with ZnO to serve as a seed layer. This coated graphene was immersed in a Zn precursor solution to grow ZnO nanotubes by the hydrothermal method [174].

A slightly different sensor consisting of ZnO/Al_2_O_3_ nanofibers, Au *np* and graphene/chitosan has been prepared for simultaneous detection of catechol and hydroquinone, two phenolic isomers widely used in diverse industries that can be harmful to humans and the environment. The limits of detection were 3.1 μM for catechol and 0.19 μM for hydroquinone, also showing separation in the detection of both isomers. The ceramic fibers were processed by electrospinning and electrodeposited together with Au nanoparticles on a graphene-chitosan-coated carbon glass electrode. This complex electrode gave the best oxidation potentials and separation for both compounds by using differential pulse voltammetry detection tools [175].

#### 4.1.5. CuO/rGO Sensors

A CuO/rGO layered composite has been prepared for the detection of CO at RT. Flower-like clusters of CuO *np* were synthesized by hydrothermal reaction and autoclave treating at 180 °C for several hours. A circuit board with interdigitated imprinted Ni/Cu electrodes was used as a substrate where the CuO and GO layers were sequentially deposited by successive immersion in corresponding solutions of respective polyelectrolytes of opposite charge. The sensor was tested for CO concentration in the range 0.25–1000 ppm, showing a low detection limit of 1 ppm and low recovery times, but above all, the RT working conditions were a distinct advantage [176].

In a different work, a CuO/rGO hybrid sensor was fabricated by the hydrothermal method for the non-enzymatic detection of glucose, which overcomes the problems of using glucose oxidase. The one-pot hydrothermal method was employed to synthesize the nanocomposite powders from GO, a Cu salt and urea. After autoclave treatment at 180 °C, freeze-drying and calcination at 300 °C, the N-doped graphene aerogel appeared decorated with CuO nanoparticles. This mixture was drop cast (water vehicle) over a glassy carbon electrode for the amperometric detection tests. The sensor was tested for the accurate detection of glucose in human fluids [177]. 

The fabrication of a sensor of interest for high blood pressure patients based on a CuO/rGO nanocomposite for the simultaneous detection of cholesterol, uric acid and ascorbic acid has been quite recently reported [178]. The composite material was synthesized from precursors (GO and copper acetate). The working electrode for the electrochemical sensing was prepared by mixing the CuO/rGO powders with an ionic liquid, graphite powder and paraffin oil. The sensor was successfully tested in human fluids.

#### 4.1.6. Fe_2_O_3/_GO Sensors

Hematite is an n-type semiconductor material that has also been used as a gas sensor. Composites of α-Fe_2_O_3_ and GO showed a comparatively enhanced detection performance against ethanol gas than hematite *np* alone. Composites were prepared from an aqueous suspension of GO and ferric chloride hexahydrate that was reacted with ammonia under stirring, autoclave treated at 100 °C for 12 h, and separated by centrifugation. The sensor was fabricated by coating a small ceramic tube with the Fe_2_O_3_/GO composite using water as a vehicle, while attaching gold electrodes and wires, and introducing into the tube a Ni–Cr alloy coil to serve as the heating element. The whole system was kept at 200 °C for several days for stabilization. This system, and in particular the 8:1 (Fe_2_O_3_:GO) composition, showed a high sensitivity for ethanol detection in the range of 50–600 ppm at the working temperature of 260 °C [179].

Following a very similar hydrothermal synthesis route to the above, Fe_2_O_3_/rGO composites with variable rGO contents (0.5–4 wt.%) were processed and used for coating a thin alumina tube, similarly as just described. This sensor was placed into an airtight container with two chambers that were filled with air and the analyte gas. This sensor demonstrated high sensitivity to acetone but also exhibited good performances against methanol and ethanol exposures. The response of this sensor was measured as the rate between the respective resistances, indicating an enhanced response at a working temperature of 225 °C. The sensor proved long-term stability (up to 20 days) and short time response (2s), which the authors attributed to the multiple p-n heterojunctions between Fe_2_O_3_ and rGO [180]. Some images of the microstructure of a α-Fe_2_O_3_/GO hybrid processed by the hydrothermal route are displayed in Figure 10b.

Following a different solid-state route, Fe_2_O_3_/rGO composites were obtained by first reacting FeCl_3_·6H_2_O, NaOH and sodium lignosulfonate (surfactant with many hydrophilic groups) in an agate mortar, and subsequent ethanol washing and centrifugation steps to achieve FeOOH powders that were calcined at different temperatures (300–600 °C). Separately, GO was prepared by the Hummers method and reduced by using hydrazine. The sensor was fabricated by coating a previously etched and cleaned glassy carbon electrode with a paste of graphene and Fe_2_O_3_ (2 mg/3 mg) in 0.4 mL of a chitosan water solution. Detection tests were done in an electrochemical cell of three electrodes (the working Fe_2_O_3_/rGO electrode plus the counter and reference electrodes). The electrode with 1.5 wt.% of lignosulfonate calcined at 400 °C displayed the best sensing performance for H_2_O_2_ detection, with a detection limit of 1.1 μM, linear response in the range 1–6000 μM, and short response time (<2 s) [181].

### 4.2. Other Ceramic/Graphene Hybrid Sensors 

#### 4.2.1. Al_2_O_3_/Graphene Sensor 

A hybrid of graphene and alumina nanofibers was used as a sensing material for the detection of ascorbic acid. The heterostructure was formed by graphene foliates grown along aligned γ-Al_2_O_3_ nanofibers of 7 nm diameter by a free-catalyst CVD process. The working electrode was prepared by crushing the nanofibers and dispersing them in an isopropanol-Nafion-water solution, which was drop cast over a glassy carbon electrode to form a layer of 30 μm. Electrochemical measurements revealed high sensitivity and selectivity for sensing ascorbic acid at a determined concentration of graphene foliates in the nanofiber [182].

#### 4.2.2. BaTiO_3_/rGO Sensor 

The nanocomposite material of BT and rGO was used for detecting ractopamine, a food toxic that has been used for a long time in food processing for preventing spoiling. However, the limits of this chemical in meat have been put at 5 mg/kg because of possible cancer interactions. The BT *np* prepared from chemical precursors were mixed with GO sheets and reduced by ultrasonication. After centrifugation and drying, the mixture was dispersed in ethanol and the paste was screen printed on a glassy carbon electrode. The detection of the toxic through differential pulse voltammetry indicated high sensitivity with a detection limit of 1.52 nM and linear response up to 500 μM. At the same time, low interference with other biomolecules such as dopamine uric acid, ascorbic acid, folic acid glucose was proved [183].

#### 4.2.3. MnO_2_/CNT/Graphene Sensor

**Figure 11:**
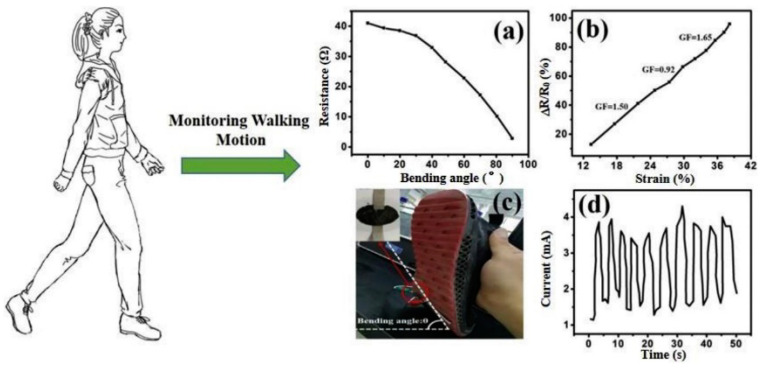
(**a**) Plots of the resistance of a strain-sensitive MnO_2_ *np*/CNT/GO film at various bending angles and (**b**) resistence changes with strain. (**c**,**d**) A photograph of a sensor attached to a sole and the corresponding current signals for detecting the walking motion of a human, reprinted from reference [184], copyright (2020), with permission from Elsevier.

A hybrid and flexible film with capability as strain sensor was prepared by hydrothermal synthesis of CNT and MnO_2_; the resulting MnO_2_ *np*/CNT powder was mixed with a GO solution, filtered to prepare flexible films, freeze-dried and treated at 900 °C for N-rGO doping. The resistance of this sensor decreased from 42 to 3 Ω when the bending angles were increased from 0 to 90, and was tested for monitoring human walking (Figure 11) by attaching the sensor to the surface of a shoe [184].

### 4.3. Ceramic/Graphene Bio-Sensors for Virus Detection

Recently, the research on sensing methods for different viruses has intensified as can be seen by recent reviews on the subject [185]. Focusing just on ceramic/graphene biosensors for virus detection, the examples are relatively few; we can highlight an optical sensor that uses surface plasmon resonance spectroscopy for detection of a protein of the dengue virus. The sensor consists of a thin film of cadmium sulfide (CdS) quantum dots and amine-functionalized GO, coupled to a specific monoclonal antibody, deposited over an Au-coated glass slide. The CdS-NH_2_GO thin films showed a quite low detection limit of 0.001 nM for the E-protein of the dengue virus [186].

Within this class, we can mention the first commercial graphene biosensor chip mounted on a ceramic package (called AGILE) for the detection of viruses and different small molecules in mostly serologic tests. The sensor was made of CVD-graphene grown on Cu films that were transferred to Si wafers and passivated by a SiO_2_ coating, the chip is readily fitted into a commercial packaging and connected to the adequate hardware for the analysis. Similar effectiveness to the plasmon resonance spectroscopy is claimed [187]. However, the ceramic part of the latter sensor does not play any active role in the detection itself. 

**Figure 12:**
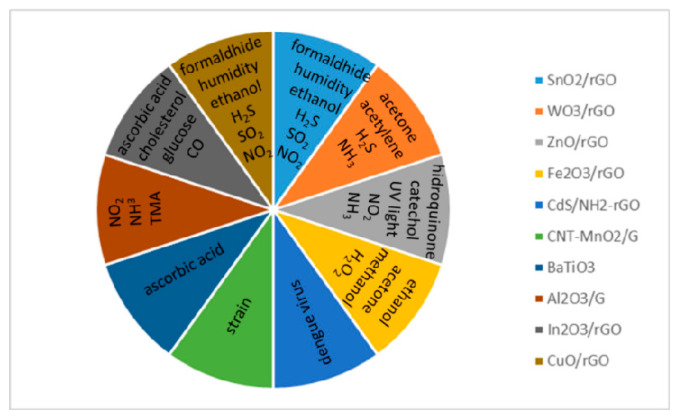
A chart summarizing the main types of ceramic/graphene-based sensors with the indication of the different tested analytes.

A pie chart summarizing the main ceramic/graphene nanocomposites sensing materials and corresponding analytes referred to in the review is depicted in Figure 12.

## 5. Ceramic/Graphene Composites for Electromagnetic Interference Shielding

Nowadays, electromagnetic interference (EMI) pollution is continuously increasing because of the strong development of communication technologies and electronic devices. The shielding efficiency (SE) is defined as the reduction in the magnitude of the incident electromagnetic field upon transition across a material, which takes place by reflection, absorption and multiple internal reflections. For many applications, the test sample should reflect as low energy as possible while maximizing absorption. Materials with dispersed conductive fillers show reflection as the primary shielding mechanism and absorption as secondary, but the shield should have electric and/or magnetic dipoles that interact with electromagnetic (EM) fields in the incident radiation to reduce radiation reflection loss and improve absorption. Ferromagnetic or ferrimagnetic materials would be a possible solution but their intrinsic cut-off frequency is usually below the low GHz range, hindering their use as EMI shielding over a broad GHz range. Therefore, broadband shielding materials that work in a wide range of frequency are currently the main research topic. Despite the great efforts already made, simultaneously obtaining minimum reflection and maximum absorption remains rather challenging. In this context, high-performance microwave absorbing materials (MAM) with small thickness, low density, wide bandwidth, and strong absorption have recently attracted great attention in solving EMI pollution. In this field, ceramics may have a significant role, particularly for high-temperature applications and harsh environments, as they are chemically inert, wear-resistant, thermally stable and strong. Silicon carbide materials (fabricated directly by sintering SiC powders or by infiltration with PDC) stand out owing to their light weight, semiconducting behavior, high dielectric loss and extremely good oxidation resistance at high temperature [188]. The PDC route is interesting because those polymers can be designed on a molecular level allowing modifications for a fine-tuning of properties, and also favors a better dispersion of second phases and permits development of singular structures. 

Recently, 2D layered conductive materials with abundant interfaces have attracted great interest in the field of microwave absorption and EMI shielding. Particularly, graphene has received special attention due to its low density, high specific surface area, strong dielectric loss, and high electronic conductivity. However, to address the interfacial impedance mismatching of single graphene materials, ceramic/graphene composites have been broadly considered [189,190]; among the factors affecting their EMI shielding are the absorber type and content, thickness, and preparation method. For practical applications of ceramic/graphene composites, the main objective is to achieve good EM performance (including wide effective bandwidth, low reflection coefficient (RC) or large SE, and high-temperature stability) with minimum filler content and the tiniest thickness. Accordingly, good filler dispersion and interfacial interaction are crucial aspects for a larger interfacial polarization because decrease the percolation threshold while increasing the contact area with the ceramic matrix. 

There is much work on the development of advanced ceramic/graphene composites for EMI shielding, focused on EM properties in the X-band (8.2–12.4 GHz) for military and communication applications, and the Ku-band (12.4–18 GHz) for small aperture terminal systems or the K-band (18–26.5 GHz) for intelligent transportation systems and vehicle radar, see for example two recent reviews [189,190]. In general, the enhanced EM performance in these composites is ascribed to conduction losses through the graphene filler network, multiple internal reflections at interfaces, and dielectric loss of the induced micro-capacitors at graphene/ceramics/graphene ensembles, including interfacial polarization due to space charge storage. Superior EMI shielding performance has been demonstrated for rGO compared to graphene due to an enhanced polarization loss associated with relaxation phenomena of functional groups and defects. Main results on ceramic/graphene composites with high EMI shielding and microwave absorbing performances are collected in Table 2 and Table 3, respectively, and they will be presented in the next sections according to their structure, i.e., composites densified by SPS and HP, synthesized from ceramic precursors, hierarchical light structures, and multicomponent systems.

### 5.1. Ceramic/Graphene Composites Sintered by SPS and HP

Most of the materials evaluated in the last four years were dense, sintered using uniaxial loads (SPS or HP techniques), to promote the orientation of the graphene fillers and the formation of the microcapacitor network. For example, in highly aligned SiC/GNP composites fabricated by HP at 1900 °C [191], absorption is the dominant contribution to the total EMI shielding; in these composites, the SE in the Ku band increased with the GNP content to more than 40 dB at 3 wt.%, and then decreased to ~31 dB for 5 wt.% GNP, probably because of sheet agglomeration. Highly dense Al_2_O_3_/GNP, B_4_C/GNP and MgO/GNP composites fabricated by HP have also shown great potential for microwave absorption applications [192,193,194], enhanced electrical conductivity, good microwave absorption, and EMI shielding performance in the X-band up to 400 °C (Table 2). The real permittivity, imaginary permittivity and dielectric loss tangent increased with GNP content. For Al_2_O_3_, the highest EMI SE was observed for 2.0 vol.% GNP, with EMI SE ~23 dB in the whole X-band at 1.5 mm composite thickness, this value increased up to 37.4 dB at 8.2 GHz when the temperature increased from 25 to 400 °C [192]. B_4_C/GNP composites showed a high EMI SE of 40 dB with 2 vol.% GNP at a sample thickness of 1.5 mm, which persevered at 800 °C (35 dB) [193]. In the case of MgO/GNP composites, the minimum RC of −36.5 dB at 10.7 GHz was observed for 2.5 vol.% GNP at 1.5 mm thickness [194].

**Figure 13:**
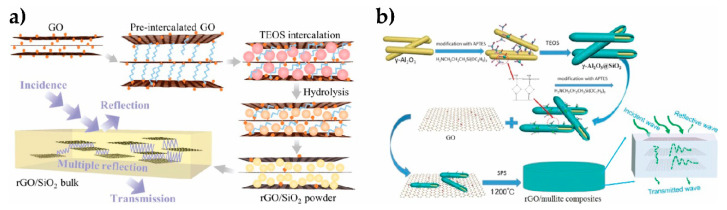
Schematics of the procedures for preparing (**a**) SiO_2_/rGO and (**b**) mullite/rGO composites for EMI applications, adapted with permission from references [195], copyright (2020), American Chemical Society and [196], copyright (2018), American Chemical Society, respectively.

The SPS method has also been used to develop highly anisotropic ceramic/graphene composites. For example, Huang et al. [195] used a molecular level intercalation approach to mix rGO sheets and silica nanolayers, and SPS densification. In this way, rGO sheets were highly aligned inside the silica matrix with the ab plane perpendicular to the loading axis (Figure 13a). The peculiar microstructure improved the EMI SE values (29–33 dB for 2–6 vol.% rGO) in the X-band by multiple interlayer reflections. On the other hand, mullite/rGO composites have been fabricated by SPS at 1200 °C but using core–shell structured γ-Al_2_O_3_@SiO_2_ powders as mullite precursors (Figure 13b) [196]. With this strategy, very low rGO contents (0.89 vol.%) produced composites with anisotropic electrical conductivity (in-plane values of 696 Sm^−1^ and one order of magnitude lower cross-plane values), which presented a very large loss tangent and EMI SE (>32 dB) in the K band. 

### 5.2. Ceramic/Graphene Composites Synthesized from Precursors 

As shown in Table 2 and Table 3, the PDC route is one of the most recently used methods for developing ceramic/graphene composites for EMI shielding applications. A comparison of the effect of GNP and MWCNT on the X-band microwave absorption properties of silicon carbonitride (SiCN) ceramics proved the better performance of GNP fillers [197]; minimum RC at 3 mm of −54 dB for SiCN/GNP compared to −48 dB for SiCN/MWCNT, with corresponding effective absorption bandwidth (EAB) of about 1.5 GHz and 0.9 GHz, respectively. Liu et al. [198] prepared SiCN/rGO nanocomposites, from polysilazane and GO, which exhibited an exceptional minimum RC of −62.1 dB at 9.0 GHz, and the EAB of 3.0 GHz at a thickness of 2.1 mm for a GO content of 2.5 wt.%. Similarly, the 12 wt.% GO composite showed good shielding effectiveness (SE = 41.2 dB) for a thickness of 2.0 mm. Song et al. [199] reported a SiBCN/rGO composite with 10 wt.% GO that showed RC of −34.56 dB and EAB of 2.46 GHz in the X-band, at the thickness of 1.8 mm. When the composite was annealed (at 1700 °C), SiC nanocrystals precipitation and the numerous interfaces improved RC and EAB to −46.73 dB and 3.32 GHz, respectively. 

Despite the number of current studies on the outstanding EM performance of ceramic/graphene composites, analysis on high-temperature EM properties are scarcer, because of graphene’s poor stability, particularly above 600 °C in oxygen-rich atmospheres. However, there are some recent studies [192,200,201], for example, EM properties of SiC/graphene-like C composites synthesized from PDC showed high and stable EMI SE both at RT and at 600 °C in the air [200]; the highest EMI SE (36.8 dB at RT and 33.8 dB at 600 °C) was obtained for the 7.6 wt.% carbon composite. Luo et al. [201] explored the EM properties of SiBCN/rGO@Fe_3_O_4_ composites after oxidation at 600 °C for 2 h. Graphene@magnetic *np* were proposed to decrease impedance mismatch between the air and the graphene material, and simultaneously increase the magnetic loss. The SiBCN/rGO@Fe_3_O_4_ composite processed by PDC technology showed excellent EM properties with minimum RC of −43.8 dB and EAB of 3.4 GHz; after oxidation at 600 °C, the composites exhibited better EM wave absorption, where the RC decreases to −66.2 dB and EAB increases to 3.7 GHz in X-band. Besides, the composites were stable at high temperatures (up to 1100 °C) in both argon and air, owing to the protecting effect of the ceramic matrix around the rGO@Fe_3_O_4_ inclusions.

### 5.3. Ceramic/Graphene Hierarchical Light Structures

Structures with a large specific area and many interfaces, such as foams, multicomponent systems or other hierarchical structures can also enhance SE. Accordingly, some attempts have been done on 3D graphene aerogels and foams due to their high porosity (lightweight), large specific surface area (increases the number of reflections and scatterings) and improved dispersion of graphene sheets, which yields longer pathways for rapid attenuation of EM wave. Moreover, these 3D graphene structures have excellent mechanical characteristics such as compression/recovering capability. Finally, they offer the possibility of anchoring different nanoparticles/nanowires to the graphene surface, for further increases of the impedance matching and interfacial polarization, and to promote additional synergistic loss effects [202]. Lightweight 3D SiBCN/graphene composites were synthesized by low-pressure CVD/CVI of a graphene foam (GF) [203], which exhibited low density (0.64 g·cm^−3^) and SE of ~19 dB, with corresponding specific shielding effectiveness (SE divided by density) of 29 dB·cm^3^·g^−1^. A SiC protective coating over SiBCN/GF composites slightly increased SE to 20.8 dB, which remained practically unchanged up to 1100 °C (93% SE at RT) [204]. In a different work, 3D SiBCN/graphene composite with good EM wave absorption properties was in-situ prepared by mixing polyborosilazanes and sugar as the carbon source [205], giving RC of −24.24 dB and EAB over 10 dB of 5.2 GHz at a thickness of 2.5 mm. Yang et al. [206] developed 3D SiC/CNT-GF composites by in-situ growing CNT on a GF, and subsequent infiltration with polycarbosilane, displaying improved EMI SE (32.1 dB) in X-band with a very low density of 0.95 g/cm^3^ (specific SE of 33.8 dB cm^3^·g^−1^). The porous structure ensured the penetration of incident EM waves and facilitated dissipation by multiple reflections.

**Table 2:** EMI shielding performance of ceramic/graphene composites, arranged by type of graphene filler.

Filler	Filler Content	Ceramics	Thickness (mm)	σ (S·m^−1^)	EMI SE (dB)	Frequency Range	Ref.
GNP	3 wt.%	SiC	3	100	43	Ku	[191]
GNP	2 vol.%	B_4_C	1.5	1850	40	X-band	[193]
G-like	7.6 wt.%	SiC	2.8	0.13	36.8	Ku	[200]
rGO	0.89 wt.%	mullite	1.4	696	32	K	[196]
GNP	2.0 vol.%	Al_2_O_3_	1.5	120	23	X	[192]
rGO	2–6 vol.%	SiO_2_	2	100–1472	29–33	X-band	[195]
rGO	12 wt.%	SiCN	2	-	41.2	X-band	[198]
rGO-CNT	15 wt.%	SiCN	2	5.7	67.2	X-band	[198]
3D-CNT-GF	n.a.	SiC	2.5	224	32.1	X-band	[206]
3D-GF	0.5 wt.%	SiBCN	1.3	-	19	X-band	[203]

### 5.4. Graphene-Based Multi-Component Systems 

**Figure 14:**
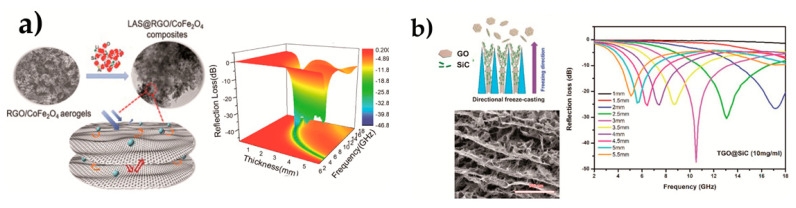
Microwave absorption performance of (**a**) LAS/CoFe_2_O_4_/rGO composites. Reprinted from [209], Copyright (2019), with permission from Elsevier; and (**b**) SiC@rGO aerogels. Reprinted from [213], Copyright (2017) with permission from Elsevier.

Regarding graphene-based multi-component systems, Han et al. [207] reported on SiOC/rGO structures with in-situ grown SiC nanowires (SiC_nw_) that displayed an outstanding EM wave absorption ability with an *RC* of−69.3 dB for 3 wt.% rGO. This high value was attributed to the conductive network, SiC_nw_ stacking faults and rGO defects. Recently, Song et al. [199] combined PDC and chemical vapor infiltration (CVI) to fabricate SiBCN/rGO-SiC_nw_ composites in which SiC_nw_ (2.29 wt.%) and rGO (0.5 wt.%) overlapped, forming a conductive pathway that significantly enhanced dielectric property and EM absorption characteristic, even at high temperatures and in harsh environments. The minimum RC was −42.02 dB at a thickness of 3.6 mm with a corresponding EAB of 4.2 GHz. Permittivity increased with temperature up to 400 °C because of the enhanced charge carrier density, and decreased above that temperature due to rGO oxidation attaining at 600 °C a minimum RC of −39.13 dB with an EAB that covered the entire X-band. The SiBCN/rGO-SiC_nw_ composite maintained good EMW absorption properties after oxidation at 900 °C, with a minimum RC of −10.41 dB. On the other hand, SiCN/rGO-CNT composites were obtained from a single-source precursor containing chemically bonded GO-CNT hybrids [198]. For the 15 wt.% GO-CNT (GO:CNT ratio of 4:1 in wt.%) composite with a sample thickness of 2 mm, an outstanding SE of 67.2 dB was obtained. Liang et al. [208] synthesized a dielectric-magnetic nanocomposite from hybridized 3D CoFe_2_O_4_/rGO nanoparticles by simple hydrothermal and freeze-drying routes. The amount of Co^2+^ and Fe^3+^ allowed adjusting the permittivity of the CoFe_2_O_4_/rGO hybrids that achieved RC of −38 dB and EAB of 5.9 GHz for a thickness of 2.2 mm. Enhanced absorption performance was achieved for a CoFe_2_O_4_/rGO hybrid coated with wave-transparent material lithium–aluminum–silicate (LAS) glass-ceramics by the sol-gel method Figure 14a [209], reaching a minimum RC of −50.0 dB at a sample thickness of 2.3 mm, and the EAB of 6.16 GHz for 2.0 mm. This outstanding performance was ascribed to a remarkable impedance matching and multi-dipole polarization. Similarly, Fe_3_O_4_@LAS/rGO composites with extraordinary electromagnetic wave absorption performance have been developed [210] from Fe_3_O_4_ nanospheres (200 nm) attached by LAS nanoparticles and distributed among the graphene sheets. The EM performance depended on the LAS content, reaching for the Fe_3_O_4_/LAS molar ratio of 1/0.2, RC values of −65 dB at 12.4 GHz with a thickness of 2.1 mm and the EAB with RC values less than −10 dB (over 90% EM wave absorption) was up to 4 GHz. Compared to Fe_3_O_4_/rGO composites, the addition of LAS significantly enhanced the absorption effect of the material in the X-band and Ku-band (8–18 GHz) and accordingly the thickness of the sample decreased.

Ye et al. [211] grew edge-rich graphene via CVD in porous Si3N4 ceramics, thus reporting tailored structures and tunable dielectric properties. This material exhibited superior EM wave absorption with very low graphene filling (<0.01 wt.%), as compared to common CVD graphene and rGO-based materials, achieving RC of −26.7 dB and EAB of 4.2 GHz covering the entire X band, for a thickness of 3.75 mm. On the other hand, a sandwich-like hierarchical structure of Si3N4/rGO was attained through in situ growth of Si3N4 by CVI on a GO foam obtained by directional freeze-drying [212]. These Si3N4/rGO composites demonstrate unique temperature-independent dielectric properties and EM performance; at absorbent content of 0.16 wt.%, the EAB covered the whole X-band at broad sample thicknesses (4.3 to 4.6 mm) and temperature range (up 600 °C). We can also cite the work by Jiang et al. [213] on SiC/rGO aerogels fabricated by directional freeze-casting from dispersions of GO-coated SiC whiskers. In this way, lightweight (72 mg⋅cm^−3^) hierarchically ordered aerogels of rGO wrapped SiC whiskers with high microwave absorption performance were developed (Figure 14b). The minimum achieved RC was −47.3 dB at 10.52 GHz at 3 mm, and EAB reached 4.7 GHz.

**Table 3:** Microwave absorbing performance of ceramic/graphene composites.

Filler	Filler Content (wt.%)	Matrix	Thickness (mm)	min RC (dB)	EAB (GHz)	Ref.
GNP	2.5 vol.%	MgO	1.5	−36.5	-	[194]
rGO	2.5	SiCN	2.1	−62.1	3	[198]
GNP	1	SiCN	3	−54	1.5	[197]
rGO	10	SiBCN	1.8	−34.6	2.5	[199]
rGO@SiC_nw_	3	SiOC	2.35	−69.3	3.9 (400 °C)	[207]
rGO@SiC_nw_	0.5 rGO/2.3 SiC_nw_	SiBCN	3.6	−42	4.2	[199]
rGO@Fe_3_O_4_	0.3	SiBCN	2.15	−43.8 (RT)	3.4 (RT)	[201]
				−66.2 (600 °C)	3.7 (600 °C)	
3D Gr-like	-	SiBCN	2.5	−24.2	5.2	[205]
graphene	<0.01	Si_3_N_4_	3.75	−26.7	4.2	[211]
3D SiC/rGO	0.15 GO: 1.0 SiC	SiC	3.0	−47.3	4.7	[213]
3D CoFe_2_O_4_@rGO	n.a.	-	2.2	−38	5.9	[208]
3D CoFe_2_O_4_@rGO Fe_3_O_4_@rGO	n.a. n.a.	LAS LAS	2.3 2.1	−50 −65	6.16 4.0	[209] [210]

## 6. Catalytic Applications of Ceramic/Graphene Composites

Nowadays, graphene is increasingly becoming an alternative to metal-based catalysts, strategic materials currently subjected to high costs and limited availability. This carbon allotrope offers numerous advantages, notably low production cost, low toxicity, very high surface area and unique tuneable surface chemistry, which enables its use in photocatalysis, electrocatalysis and carbocatalysis [214]. Recently, research is mainly looking for new ceramic/graphene catalysts for contaminants removal or for the production of hydrogen, using either metal or metal-free catalysts.

### 6.1. Catalysts for Contaminants Removal

Initial works on ceramic/graphene composite catalysts were published in 2015 by Vinothkannan et al. [215] and Zhao et al. [216], where Fe_3_O_4_/rGO [215] and Fe_3_O_4_/rGO/NiO [216] composites were developed for the successful photocatalytic degradation of dye molecules, such as methylene blue (Fe_3_O_4_/rGO), p-nitrophenol and rhodamine B (Fe_3_O_4_/rGO/NiO). The large surface area, adsorptive efficiency and numerous catalytic active sites provided by rGO enhanced the degradation of these dyes. Interestingly, all works reported until now have selected GO as the source of graphene-based catalyst, in part attributed to its lower production costs, which are often chemically or thermally reduced to rGO to improve the carrier mobility and electrocatalytic properties.

**Figure 15:**
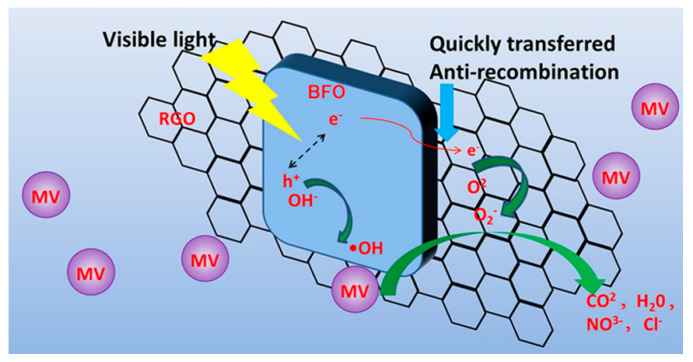
Schematic illustration of the photocatalytic mechanism for the degradation of methyl violet (MV) solutions using BFO/rGO composites. Reprinted from [217], Copyright (2017) The American Ceramic Society, with permission from John Wiley and Sons, Inc.

The addition of rGO to photocatalytic ceramics like Bi_2_Fe_4_O_9_ (BFO), TiO_2_ or BiVO_4_/SiO_2_ has considerably improved those processes. For example, a BFO/5 wt.% rGO composite increased the degradation efficiency of methyl violet solutions [217], which authors attributed to a mechanism of electron-hole pair formation in BFO under visible light irradiation (Figure 15) and fast transfer of photoelectrons to the rGO surface that lead to the oxygen reduction, while holes on the BFO surface oxidized the methyl violet. In the case of TiO_2_/graphene composites, Li et al. [218] found that a TiO_2_/8 wt.% of rGO composite loaded onto honeycomb ceramic plates via the sol-gel method showed excellent performance for removing clothianidin in pesticide wastewaters. Salem et al. [219] developed TiO_2_/rGO decorated onto mullite rods to recover wastewaters contaminated by the cationic dye. Trinh et al. [220] synthesized BiVO_4_/SiO_2_/GO nanocomposites by the hydrothermal method, and reported a much higher efficiency for methylene blue removal than the plain ceramics due to the considerably augment of the specific surface area and pore size promoted by GO sheets. There is currently a new line in the catalysis research area that looks for embedding the catalytic species into 3D printed structures because the control over the size, shape and porosity of the structures, as can be seen in Section 10.

Regarding metal-containing catalysts, Khojasteh et al. [221] produced rGO/TiO_2_/Pd-Ag composites by combining hydrothermal and photo-deposition processes. The degradation of rhodamine B and methylene blue under ultraviolet light irradiation was favored by rGO sheets, which behaved as an electrically conductive pathway for the photogenerated electrons and also as a good adsorbent for organic dye molecules. Similarly, enhanced catalytic performance for the degradation of methyl orange was reported by Wang et al. [222] using an Ag/TiO_2_/rGO composite catalyst.

### 6.2. Catalysts for Hydrogen Production

Ceramic/graphene catalysts have recently been employed in a new application as the electrocatalytic hydrogen evolution reaction (HER). In this way, Mondal et al. [223] synthesized uniformly dispersed thin and small MoS_2_ nanosheets decorated on rGO. The composite containing 20 wt.% of rGO exhibited a superior and more stable electrochemical HER performance than the monolithic material. In fact, the rGO improved the electrical conductivity and accelerated the electron transfer process, which led to smooth and superior kinetics. Zhang et al. [224] obtained high visible light catalytic hydrogen production activity and excellent stability when used TiO_2_/MoS_2_/1 wt.% GO photocatalysts. Hanniet et al. [225] developed electrocatalytic composites of SiBCN PDC and rGO, which showed promising HER activity by varying the nitrogen, boron and oxygen content in the PDC, while rGO acted as the porous conductive network and allowed the fast transport of the charge carriers to activate the ceramic.

## 7. Ceramic/Graphene Composites in Biomedicine

Bioceramics have long been used in bone tissue engineering (BTE) applications [226,227]. Due to their inorganic nature and rigid mechanical response, these materials are traditionally applied to hard tissue repair, such as bone and teeth, although their potential to regenerate various types of damaged soft tissues has also been evidenced. Bioinert ceramics, such as alumina, zirconia, silicon carbide, and silicon nitride provide good mechanical support without interacting with the surrounding tissues. On the other hand, bioactive ceramics mainly include calcium phosphate-based materials which have a similar composition to the mineralized bone, i.e., hydroxyapatite (HA), β-tricalcium phosphate (β-TCP), and calcium phosphate cement; they may interact with surrounding tissues with strong osteoinductive responses, promoting the formation of new bone tissues. Accordingly, they are excellent scaffolding materials for bone tissue regeneration. In particular, HA has been widely used as bone grafts in orthopedic or maxillofacial surgery. Likewise, bioactive glasses such as calcium and phosphate-containing silica glasses, usually used in orthopedics and dentistry, have been recently explored for BTE applications, since they present good biocompatibility and could produce HA and silica compounds, thus promoting cell differentiation and osteogenesis. Nevertheless, their slow resorbing rate by the surrounding tissues limits their use; besides, the inherent brittleness and low strength of bioceramics are major issues preventing their employ in stress-bearing areas. 

Owing to their nanoscale size, excellent mechanical properties, good electrical conductivity, large specific surface area, tunable surface functionalities, photoluminescence properties, high biocompatibility and antibacterial activity, GRM possess enormous potential in biomedicine for BTE, drug and gene delivery, biosensors, bioimaging applications, antibacterial agents, and photothermal therapies [228,229,230]. Biomedical applications of GRM represent 37.4% of their total applications according to Banerjee et al. [231]. GRM have been added as reinforcing fillers in bio-scaffolds with the aim of combining the biocompatibility characteristics provided by bioceramics with the notable physical properties of graphene. As conductive materials, GRM increase cellular activity and encourage bone tissue repair, while display good antibacterial activity [232]. In fact, GO has an excellent ability to interact and adhere to cells and other biomolecules, consequently, supporting cell viability and promoting osteogenic differentiation; while surface-modified GO presents a unique ability to accelerate HA mineralization. Therefore, most of the research on scaffolds for BTE applications has focused on GO and rGO. Several reviews have been recently published on this matter [228,230,233] that evidence the extensive work done. 

Despite the advantages in the bone repair application, some concerns remain about potential toxicity and possible in vivo residues. Current studies demonstrate that toxicity depends on graphene size, being bigger for smaller sizes (<100 nm) than larger ones (>400 nm), and concentration (above 10 μg⋅mL^−1^ might inhibit proliferation of bone marrow mesenchymal stem cells). On the other hand, graphene’s potential toxicity may be decreased through functionalization and compounding with other materials, as the potential to penetrate the cell membrane would be reduced. Many recent studies have been conducted to investigate the potential toxicity of GRM in their interaction with cells and tissues, in vitro and in vivo experiments, that being a critical issue in order to identify suitable candidates for clinical applications, see reviews [228,229,230].

### 7.1. Ceramic/Graphene Structures

**Figure 16:**
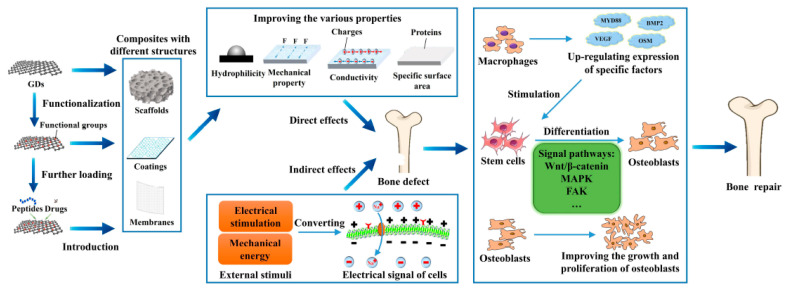
Scheme showing the possible role of graphene-based materials (GDs) in BTE applications. Reprinted from reference [234].

Composite structures shaped either as 3D porous scaffolds, 2D planar coatings or barrier membranes have been developed (Figure 16) to enhance the bone volume in bone defect zones [234]. The potential of graphene and GRM for the differentiation of various types of stem cells has captured remarkable attention [228]; actually, this ability has been ascribed to its peculiar noncovalent binding to different biomolecules that allows GRM to behave as pre-concentration platforms for osteogenic inducers, accelerating the differentiation of hMSC (Human Mesenchymal Stem Cells) into osteogenic cells. Composites often display favorable performance toward the proliferation and growth of L929 fibroblast cells, MC3T3-E1 pre-osteoblast cells and MG63 human osteosarcoma cells. A great diversity of fabrication methods can be found, i.e., the biomimetic mineralization process, the SPS technique, radio-frequency CVD, electrospinning, self-assembly, selective laser sintering, 3D printing, freeze-drying, etc.

**Figure 17:**
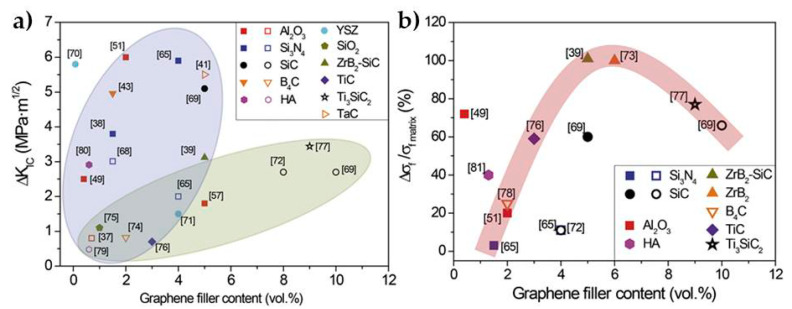
(**a**) Maximum toughness increment, and (**b**) relative strength increase data for ceramic/graphene composites (full and empty symbols correspond to GO and GNP fillers, respectively), adapted from [4], copyright (2017), with permission from Elsevier. Numbers in brackets show the corresponding references in that work. Shadowed blue area in (**a**) encircle data for GO and the green area for GNP, whereas shadowed area in (**b**) indicates the general observed trend.

The mechanical properties of bone repair materials have a significant effect on their bio-performances. It is well known that the toughening effect induced by graphene fillers in ceramic composites critically depends on their content, functionalization and dispersion degree, showing the general trend (Figure 17) of increasing up to certain content and then decreasing (for more details see review [4]). In fact, GO reinforcements generally produce higher toughness increments than GNP. Reinforcement has also been proved for bioceramics, such as hydroxyapatite, yttria-stabilized zirconia, alumina, etc. [4,235], and more recently, for bio-piezoelectric hybrid composites of barium titanate/polymethyl methacrylate [236]; in this case, the introduction of low graphene contents (<0.8 wt.%) developed materials with good biological activity and slightly higher compression strength. Reinforcement has been observed also in printed bioactive glass scaffolds containing rGO [237]. In addition to BTE applications, graphene-based composites may be applied as the contrast agent in vivo imaging for real-time detection of the bone repair process, detecting structural changes, tumorigenicity, degradation and mineralization of the bone repair materials in complex environments (see review [234]). 

The common strategy for BTE consists in simulating the natural process of bone remolding and regeneration, which can be done by using biocompatible, biodegradable, and osteoconductive or osteoinductive 3D scaffolds, which offer an optimum microenvironment to mimic the extracellular matrix in real bone tissues. The strength, stiffness and mechanical behavior of these types of scaffolds should resemble those of natural bone that shows superelastic performance with Young’s modulus of 7–27 GPa [238]. Finally, they should be porous so that they promote vascularization, present sufficient mechanical strength, and provide physical and biochemical stimuli.

Recently, 3D interpenetrating network structures were synthesized by CVD, spin-coating, and freeze-drying methods from a graphene foam and 58S bioactive glass, which revealed as promising candidates for BTE [239]. These structures showed high bioactivity in simulated body fluid (SBF) tests, facilitated the adhesion and extension of rabbit mesenchymal stem cells with higher proliferation than the plain glass scaffold, and also displayed electrical conductivity. In vivo tests confirmed that this scaffold considerably promoted the formation of new bone. In another work [232], the polymer foam replication technique was used for developing electrically conductive porous scaffolds of borate-based bioactive glass/GNP, using a dispersion of glass particles (40 vol.%) and GNP (0–10 wt.%) in ethanol. Graphene improved the electrical conductivity of the scaffolds but had a negligible effect on their mechanical properties; they were bioactive after 30 days of immersion in SBF, although the HA formation rate diminished with the graphene content, especially for shorter times. Cytotoxicity tests indicate that the MC3T3-E1 cell growth was significantly inhibited in the scaffolds containing a high amount of GNP, as compared to plain glass scaffolds. The scaffold with 5 wt.% GNP presented the best performance with an improvement of electrical conductivity, moderate cellular response and in vitro HA forming ability. Additionally, 3D printing methods have been used for developing composite scaffolds meant for bone tissue repair, which are referred to in Section 11. 

On the other hand, biomineralized HA nanocrystals/GO composites were produced by two different methods [233]; namely, by a sol-gel in situ procedure at RT from Ca(NO_3_)_2_·4H_2_O and (NH_4_)_2_HPO_4_ precursors and a dispersed GO water solution, and a biomimetic approach consisting of two soaking steps in pH-controlled environments: first, a supersaturated SBF solution to promote nuclei formation, and second, a chemically modified solution to encourage the crystallization and growth of apatite nuclei. In the HA/GO nanocomposites processed via sol–gel, spindle-like nanoparticles of HA were strongly bonded to the GO surface by means of Ca^2+^ ions that attached to the oxygen functional groups on GO. This biocomposite showed enhanced cell viability (hMSC), and induced osteoblastic differentiation without using osteogenic factors. For composites obtained by the biomimetic route, an amorphous calcium phosphate coating formed on GO sheets; these biocomposites also sustained cell viability and proliferation, but the expression of alkaline phosphatase activity was hindered.

**Figure 18:**
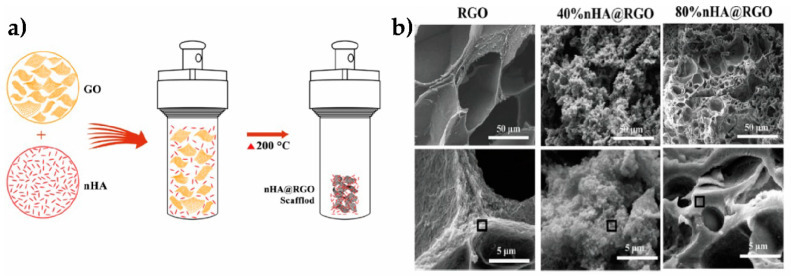
(**a**) Schematic of the self-assembly fabrication procedure for nano-HA@rGO scaffolds; (**b**) SEM micrographs of the developed materials with different amounts of nano-HA. Adapted from [241], copyright (2017), with permission from Elsevier.

The feasibility of rGO hydrogels as artificial barrier membranes for guided bone regeneration was demonstrated by Lu et al. [240]. This membrane was able to uphold the osseous space, stimulated osteo-differentiation, mineralization, and accomplished fast bone regeneration. Based on these results, several studies have reported the use of rGO to improve the properties of HA-based scaffolds. Similarly, the addition of rGO increased the scaffold mechanical performance and promoted osteogenic differentiation (see review [230]). Grafts based on HA@rGO hybrids demonstrated the ability to enhance new bone formation to a significant degree in rabbit calvarial defects, without any sign of an inflammatory response [241,242]. Moreover, in vivo experiments revealed that a 20% porous nano-HA@rGO scaffold could accelerate healing of circular calvarial defects in rabbits. These 3D porous scaffolds with different nano-HA content were synthesized by blending a GO solution and the corresponding nano-HA water suspension, followed by hydrothermal heating to induce self-assembly before the freeze-drying step, see Figure 18 [241].

### 7.2. Complex Ceramic/Graphene Composites

Organic/inorganic multicomponent hybrids of GO and HA and biopolymers (such as chitosan, collagen, or agarose) have also been developed to achieve good cytocompatibility and better hydrophilic and mechanical properties [243,244,245,246,247,248]. These composites displayed improved tensile strength and high in vitro bioactivity, enabled MC3T3-E1 cell adhesion and proliferation, and showed greater osteogenesis. Recently, various 3D porous HA-based scaffolds with bone-like organic-inorganic hybrid structures have been fabricated by different methods. For example, Fu et al. [243] prepared a nanofiber substrate of poly (l-lactic-co-glycolic acid)/HA/GO by electrospinning. The role of the polymer component (PLGA) was to provide a uniform and smooth nanofibrous 3D porous substrate with excellent biocompatibility and biodegradation properties, while GO enhanced the tensile strength and conductivity of the substrate, and HA improved the biocompatibility. Zhao et al. [246] prepared CS/nano-HA/GO particles scaffolds by an in-situ crystallization process, in which a Ca(NO_3_)_2_ and K_2_HPO_4_ aqueous solution was added to a solution of CS in acetic acid, and mixed with a GO suspension; finally, the CS/nano-HA/GO scaffolds were obtained by treating with an alkaline solution. On the other hand, a 3D porous CS/nano-HA/carbon scaffold was developed by in situ depositing HA nanoparticles on the porous carbon surface and immersion in chitosan solution before freeze-drying [247]. The freeze-drying method was also used by Liang et al. [248] to fabricate porous nano-HA/collagen (nHAC)-based biodegradable scaffolds with different amounts of GO (nHAC/PLGA/GO). Thus, collagen was first dissolved in acetic acid to form a solution, at that point, both CaCl_2_ and H_3_PO_4_ (Ca/P  =  1.66) solutions were added, then the resulting nHAC powder was collected by centrifugation and freeze-dried. This powder was added into the PLGA/GO solution, stirred and ultrasonicated for 4 h before the last freeze-drying. In another wok, the porous carboxymethyl chitosan/nano-HA/GO composite was prepared by chemical crosslinking, lyophilization and afterwards, ultrasonic washing [249]. Luo et al. used an agarose matrix mixed with N-doped graphene@HA hybrids via a hydrothermal/cross-linking/freeze-drying route [244].

Usually, graphene needs to undergo some functionalization before using in composites, scaffolds, coatings, membranes or injectable hydrogels. Properties like dispersibility and hydrophilicity can be considerably enhanced by different functional groups, thus improving the bone repair capability and the binding ability to active substances [250,251]. The surface modifications of graphene may also improve the mechanical performance, for example, Unnithan et al. developed CS/HA/GO scaffolds containing simvastatin (SV) crosslinked with GO, which showed enhanced osteogenic and biomineralization performances [252]. Recently, silica-coated rGO nanosheets, developed by a sol-gel method, were mixed with HA nanorods and densified by SPS [253]. These composites showed enhanced mechanical properties (Young’s modulus, hardness, and fracture toughness) and better biological properties (faster osteoblast-like MG-63 cells proliferation and alkaline phosphatase activity) than materials fabricated using uncoated rGO sheets.

A recent work by Udduttula et al. [254] achieved CVD graphene grown over rod-shaped nanosized powders of strontium phosphosilicate (Sr_5_(PO_4_)_2_SiO_4_, previously synthesized by a sol-gel method. In vitro studies demonstrated the deposition of a layer of needle-like amorphous apatite with alkaline phosphatase activity, calcium deposition and osteogenic differentiation ability. The composite showed no toxicity in HUVEC (Human Umbilical Vein Endothelial Cells) culture medium and significantly promoted cell proliferation.

Much more complex systems that incorporate osteogenesis-inducing drugs, peptides, proteins and anti-bacterial agents have also been developed, aiming at further improving the performance of the scaffold [252,255]. For example, Xie et al. [256] used a GO nanolayer to anchor bone morphogenetic protein-2 (BMP-2)-encapsulated bovine serum albumin nanoparticles on HA and β-TCP scaffolds, through the electrostatic interaction between the positively charged nanoparticles and negatively charged GO, in order to achieve BMP-2 sustained release for preventing a rapid degradation of β-TCP scaffolds. 

## 8. Thermal Applications of Ceramic/Graphene Composites

Many important applications of ceramic materials are linked to thermal properties and heat transfer subjects. Ceramic materials for heat dissipation and thermal management require the assistance of different properties such as electrical insulation and high thermal conductivity. However, ceramics directed to thermal insulation issues generally require very low values of both density and thermal conductivity. In any case, high thermal and mechanical stabilities remain critical attributes. Examples of thermal management use include substrates and heat sinks for light-emitting diodes (LED), computer processors and power electronic devices, as well as heat shields for automotive and aerospace applications. Thermal applications of ceramic/graphene composites also include capsules for infiltration of phase change materials (PCM) used in thermal energy storage systems; in the case of cement-based composites, these uses are reviewed in Section 9.5. Ceramic materials like SrTiO_3_ or Al-doped ZnO are used in thermoelectric-based coolers for electronic devices and batteries, then, although they could be listed in this section, they have been referred to in Section 3.2 with other thermoelectric materials. 

Within the low thermal conductivity ceramics, we can refer to aerogels and porous materials used as thermal insulation and flame retardants in buildings, motor and aerospace vehicles. In particular, low thermal conductivity materials and coatings have become essential in thermal barrier applications for gas turbines and in thermal protection systems (TPS) of Reusable Launch Vehicles for nose, wings and fin leading edges, where high reentry temperatures (900–1200 °C) and mechanical loads develop. 

GRM are considered ideal nano-inclusions for tuning the heat transfer performance of ceramics [189,257] because of their highly anisotropic thermal conductivity with in-plane and transverse values >2000 and <10 W⋅m^−1^⋅K^−1^, respectively, and remarkable properties like good mechanical strength and high electrical conductivity, which are critical for certain applications. 

### 8.1. Applications in Thermal Management

#### 8.1.1. Ceramic/Graphene Porous Structures for Heat Sinks and Thermal Energy Storage

Heat generation in power electronics is a critical matter because a rise in the device temperature can degrade its performance and even cause its failure. In thermal management applications, GRM are either integrated into the device architecture or added as filler. According to recent reviews [257,258,259], several applications envisaged for GRM and their composites refer to thermal management in LED and high power electronics, such as thermal dissipation films for mobile electronics, thermistors, and thermal heaters based on CVD graphene; which are already available in the market [259]. A single-layer graphene film in the width range of centimeters can be used as a tunable heating surface (40–120 °C) in personal-care products or clothing. Actually, graphene can be deposited on diverse substrates including curved surfaces and complex structures, which makes CVD graphene suitable for ceramic heat sinks of a large surface area. 

**Figure 19:**
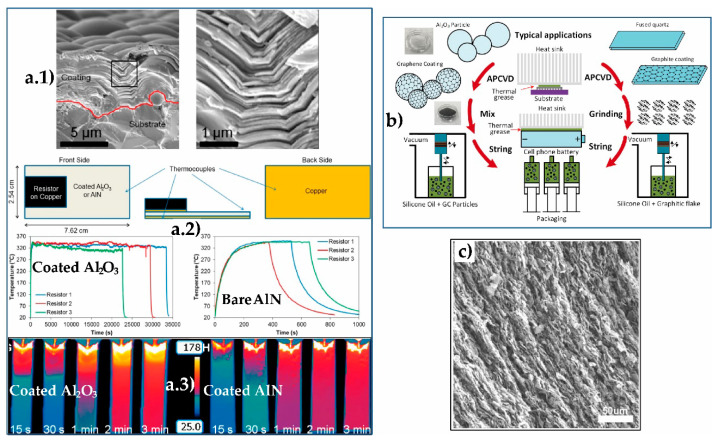
(**a**) Example of thermal management results for thick graphitic networks on porous Al_2_O_3_ and AlN ceramics processed by APCVD: (**a.1**) SEM images of the graphitic networks, (**a.2**) Schematics of the Joule heating test setup, based on a thick film resistor attached to a DBC substrate, and backside hot spot temperature profiles of both the coated Al_2_O_3_ and bare AlN DBC substrates; and (**a.3**) temperature distribution during heating of both coated substrates. Adapted with permission from reference [263], copyright (2019) American Chemical Society. (**b**) Schematics of the APCVD procedure used to develop thermal conductive fillers for silicone oil-based non-curing TIM: highly oriented thick graphitic network flakes delaminated from quartz substrates and graphitic network-coated Al_2_O_3_ spherical particles. Adapted from [265], copyright (2020) American Chemical Society. (**c**) SEM image of a highly oriented BN/rGO hybrid aerogel (GO/BN initial ratio: 1/15) adapted from reference [266]. Copyright (2017), with permission from Elsevier.

Examples of ceramic/graphene materials for thermal management applications, include architectures consisting of a CVD graphene coating on porous Al_2_O_3_ foams that display ultralow sheet electrical resistance (0.11 Ω·sq^−1^) and improved thermal conductivity (8.28 W⋅m^−1^⋅K^−1^) [260]. The incorporation of a PCM into the Al_2_O_3_/graphene foam still maintained a κ value of 2.39 W⋅m^−1^⋅K^−1^ with a latent heat of 38 J⋅g^−1^, indicating great potential for heat transfer purposes, especially for heat sink and thermal energy storage applications [261]. However, conventional CVD processes require a long time to grow nanoscale graphene films that generally show poor adhesion to the ceramic substrate, whereas micrometer-scale films with a larger thermal mass and better bonding to the substrate are needed for demanding thermal management applications such as wide band gap semiconductor devices. Recently, an atmospheric pressure CVD (APCVD) method by using dual silicon oxycarbide (SiOC) sources has been developed for depositing ∼10 μm thick graphitic networks on porous Al_2_O_3_ and AlN ceramics, Figure 19(a.1) [262,263]. The graphitic deposits on Al_2_O_3_ showed better adhesion than those obtained by conventional CVD, while delamination was detected for the AlN/graphitic coating structures. The developed structures presented sufficient thermal mass and superior thermal conductivity, 1000 W⋅m^−1^⋅K^−1^ for the in-plane and 6 W⋅m^−1^⋅K^−1^ for the cross-plane directions, measured for a 6 μm thin layer by the time-domain thermo-reflectance method [262]. The thermal management performance was checked by Joule heating using a setup based on a thick film resistor attached to a DBC substrate, as shown in (Figure 19(a.2)). The performance of the graphene-coated Al_2_O_3_ was significantly better than the displayed by AlN substrates, showing 54-fold greater resistor failure time. Besides, there was a delay in the temperature rise that helped prevent thermal shock.

#### 8.1.2. Thermal Interface Materials (TIM)

Highly efficient thermal conductivity composites that maintain electrical insulation have been developed for TIM application by using aligned graphene or boron nitride sheets [264]. The APCVD procedure was recently used to develop thermal conductive fillers for a silicone TIM [265] with a possible application as a heat sink. Two types of fillers were proved: a network of highly oriented thick graphitic flakes obtained by delamination, grinding and sieving; and graphitic network-coated Al_2_O_3_ particles (Figure 19b). The thermal conductivity of the thermal paste formed reached 4.0 W⋅m^−1^·K^−1^ when high filler contents of either type were utilized, that value was much higher than the typical commercial graphite-based thermal pastes, and very close to that of the nanodiamond-based thermal pastes. Besides, several studies have reported on 3D porous BN/graphene blends that when added to the polymeric matrix resulted in high electrical conductivity TIM [266,267] because of the graphene conducting network. An et al. [266] described the synthesis of highly anisotropic BN/graphene hybrid aerogels (Figure 19c) by hydrothermal self-assembling of a GO/BN nanoplatelet suspension. Once dried and heated at 2000 °C, the resultant BN/rGO hybrid particles were added to an epoxy resin (4.2 and 39.8 wt.% graphene and BN, respectively); the ensuing composite reached a top thermal conductivity value of ~11 W⋅m^−1^⋅K^−1^), ~72 times higher than that of the epoxy matrix. 

### 8.2. Applications in Advanced Thermal Insulation

Depending on the graphene ordering within diverse porous structures (aerogels, freeze cast scaffolds, 3D printed structures), hybrids can be used not only for enhancing thermal conduction but also for developing advanced insulating materials [264]. Differing from aerogels, freeze-cast structures with highly aligned pores show significantly better thermal insulation perpendicularly to the pore orientation. Those highly insulating materials can be applied in space systems protection and in buildings for improved energy efficiency. Some recent works refer, for example, to 3D interconnected ceramic/graphene networks with great potential as thermal insulation and flame-retardant skins [268]. This hybrid material, obtained by atomic layer deposition of Al_2_O_3_ nanoparticles on 3D graphene templates, showed very low density, superelastic response, structural robustness, high electrical conductivity, good thermal stability, flame-retardant quality and good thermal insulation (0.05 W⋅m^−1^⋅K^−1^).

### 8.3. Electro-Thermal Devices

**Figure 20:**
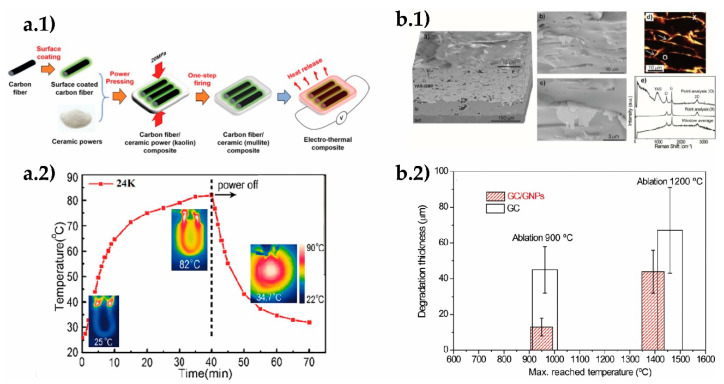
(**a**) Electro-thermal performance by Joule heating of a mullite/G-coated CF composite: (**a.1**) schematic of the processing and (**a.2**) temperature changes versus time and images of temperature distribution during testing Adapted from [269]. Copyright (2020), with permission from Elsevier. (**b**) Ablation performance of thermal sprayed glass-ceramic/GNP coatings: (**b.1**) assembly of SEM images of the top and cross-section surfaces, detail of the microstructure of the fracture surface by SEM, and Raman analysis was done on the polished cross-section Adapted from reference [271], copyright (2015) American Chemical Society. (**b.2**) The thickness of the degraded top layer versus the maximum temperature reached during ablation tests at 900 °C and 1200 °C for the glass-ceramic/GNP and plain glass-ceramic coatings. Adapted from reference [273], copyright (2016) The American Ceramic Society, with permission from John Wiley and Sons, Inc.

Graphene coatings have been used to modify carbon fibers (CF) in mullite/CF composites, in order to improve its oxidation resistance and avoid deterioration of the electro-thermal functioning under heating [269]. The processing route used by Xiong et al. [269] to fabricate ceramic/graphene-coated CF composites is shown in Figure 20(a.1), essentially reflecting how a small amount of epoxy resin was added as an adhesive thickener to a graphene-acetone solution to coat the CF, the coated CF were then added to the ceramic powder precursors (kaolin, feldspar and quartz), and the mixture was pressed and sintered at 1100 °C. This material presented significant electro-thermal qualities (rapid heating at low voltages and good temperature storage performance, i.e., slow cooling rate as shown in Figure 20(a.2), as well as good mechanical properties; accordingly, this composites deserved consideration as a potential candidate for both in-door temperature control and out-door de-icing applications.

### 8.4. Ablative Protection Systems

Ablative TPS are considered a suitable choice for aerospace applications in those cases where full heat exposure occurs for a short time [270]. Major concerns in aerospace applications are reducing weight while preserving high emissivity and very low thermal conductivity for rejection of the incoming heat flux by radiative heat transfer. Ablative composites can be applied as well in long-range ballistic missiles, high-speed brake systems and for thermal protection in engineering applications, where rapid control of heat flux threat is critical. 

Garcia et al. [271,272] developed glass-ceramic (Y_2_O_3_-Al_2_O_3_-SiO_2_ system) coatings containing GNP (1 wt.%) on SiC and SiC/CF substrates using a simple and feasible thermal spraying method (Figure 20b), which produced the orientation of the GNP plane perpendicular to the coating surface. These anisotropic composite coatings exhibited enhanced ablation resistance [273], protecting the substrate from exposure at 1350 °C for at least 30 s. The coated materials were able to withstand up to 30 heating-cooling cycles, between 1350 °C and RT, with no signs of spallation or substrate damage. This enhanced thermal performance was attributed to the high emissivity and thermal conductivity anisotropy, which favored heat propagation parallel to the surface while limited thermal conduction in the perpendicular direction, i.e., towards the substrate, restraining the generation of hot spots. Besides, as the GNP were horizontally arranged to form a carpeted network, the interaction of the hot gasses with the substrate was limited. Due to its high electrical conductivity, these coatings could also be valuable for EMI and de-icing applications.

## 9. Structural Engineering Applications of Ceramic/Graphene Composites

In this section, some applications linked to the extraordinary reinforcing effect of GRM on dense bulk and porous ceramics will be presented. Graphene fillers positively affect the mechanical properties of ceramics, in particular, fracture toughness, hardness, Young’s modulus, flexural strength, and wear behavior [4]. The addition of GRM to ceramics led to enhanced dynamic mechanical properties, damping characteristics and structural health monitoring (SHM) sensitivity, being all these properties mainly linked to the matrix/GRM interface characteristics, as it will be shown below. 

### 9.1. Tough Dense Ceramic/Graphene Composites

**Figure 21:**
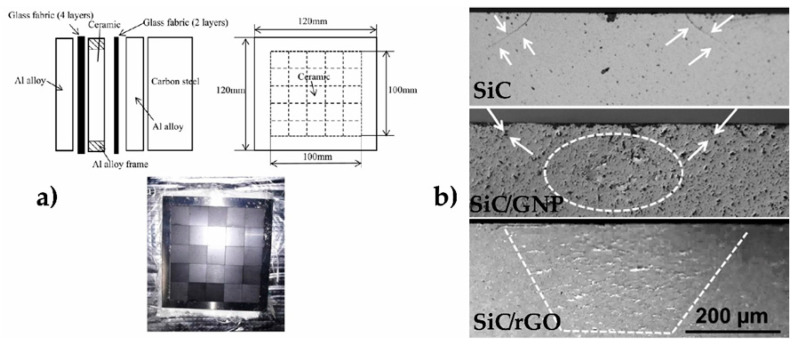
(**a**) Schematics and image of a B_4_C/GNP ceramic plate tested as armor target. Reprinted from reference [275], copyright (2019), with permission from Elsevier. (**b**) SEM images of the damage underneath Hertzian contact for monolithic SiC, and SiC/GNP and SiC/rGO composites with 5 vol.% of nanofillers. Arrows point the cone cracks whereas the dashed lines demarcate the quasi-plastic damage zones. Reprinted from reference [277], copyright (2017), with permission from Elsevier.

Fracture toughness has received specific attention as represents one of the most limiting factors for ceramics application. In particular, significant toughness increases have been reported for low graphene contents when a good filler dispersion within the matrix was guaranteed. Toughening mechanisms have been widely studied and, recently, in-situ observations of crack propagation in ceramic composites (Si_3_N_4_/rGO) inferred non-conventional crack-bridging mechanisms that combined stretching, debonding, sliding and pull-out of the piled graphene layers [274]. The rising R-curve behavior observed for ceramic/GRM composites confirmed the high reinforcement effect of graphene fillers [96], which lead to highly tolerant materials. Therefore, tough ceramic/GRM composites are viable candidates for a number of applications in ballistic armors and aerospace vehicles [189]. For example, the ballistic resistance of a B_4_C/GNP (1.5 wt.%) composite, fabricated by SPS, was significantly better than the displayed by two comparable commercial ceramic armors (SiC, B_4_C); actually, the penetration depth for a bullet speed of 818 m·min^−1^ decreased by ~45% [275]. Figure 21a shows the schematic of the B_4_C/GNP ceramic armor target plate tested. Despite graphene may obviously contribute to the development of new lightweight and mechanical resistant materials for ballistic applications, works on this subject are rare [276], possibly due to the sophisticated instrumentation required

On the other hand, the positive effect of GRM on the contact damage resistance of ceramics was investigated by Hertzian type tests, evidencing that deformation of SiC/GRM composites changed gradually from elastic-brittle to a quasi-plastic regime with the GRM content, making the composites less prone to failure under concentrated loads [277]. This behavior was attributed to shear faulting processes linked to matrix/graphene interface debonding, more evident for rGO than for GNP fillers, which led to a redistribution of stresses beneath the hard contact due to the larger number of matrix/graphene interfaces in the highly exfoliated rGO composite (Figure 21b). 

### 9.2. Ceramic/Graphene Composites for Tribological Applications 

**Figure 22:**
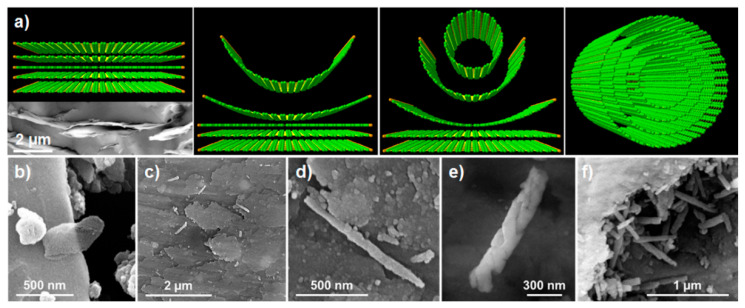
(**a**) Illustration of the roll formation in graphene stacks, (**b**) exfoliated graphene flake on the tested surface, (**c**) carbon-based tribofilm, (**d**) long carbon roll, (**e**) twisted roll formed by the combination of different rolls and, (**f**) accumulation of rolls trapped into a cavity. Reprinted from [281], copyright (2019), with permission from Elsevier.

Reducing energy waste from industrial activities, often related to friction and wear of moving parts in engineering components, is a major challenge with direct economic and environmental benefits. To enhance the tribological performance of engineering components, different strategies are investigated, such as the use of wear protecting coatings, surface texturing, new external lubricants, or the development of self-lubricant composites. Accordingly, graphene-based nanostructures have been widely incorporated into polymers, metals and ceramics, because of their excellent lubricating properties [278,279]. Most tribological studies have shown superior response of ceramic/graphene composites, with typical reductions in the friction coefficient in the 50–70% range and increases in wear resistance of few orders of magnitude, although results depend on many experimental factors, such as recent reviews on the subject reveal [4,280]. The improved tribological properties of these composites are generally associated with the formation of a self-lubricant and wear protecting carbon-rich tribofilm well adhered to the tested surface, jointly with an improved fracture toughness that would minimize wear [280]. The tribofilm is created by the sliding motion of the counter-bodies, producing the exfoliation and crushing of the pulled-out graphene fillers, which under dry testing conditions could be rolled-up, producing a roller bearing-like effect on the tribofilm top that overall reduces friction and wear [281]. Figure 22 illustrates the formation of those carbon-based rolls and the protection and lubricant tribofilm on ceramic/graphene composite tested under dry conditions.

The numerous works describing the tribological properties of ceramic/graphene composites mainly involve fundamental studies with potential uses primarily in the automotive, aerospace, and marine industries, whereas results under real testing conditions are scarcer. Accordingly, few works can be selected that focus only on specific tribological applications.

Chen et al. [282] developed a graphene-modified porous composite-bonded cubic BN grinding wheel to machine Ti-6Al-4V titanium alloys. The authors first analyzed the tribological performance of the wheel abrasive layer and workpiece using a dry pin-on-disc configuration. They found that the best flexural strength and tribological characteristics were attained when 0.01 wt.% GNP were added to the composite. Tests conducted in a grinding machine demonstrated that the addition of graphene allowed high-efficiency in deep grinding of Ti-6Al-4V titanium alloys and produced good surface quality, which probably entails opportunities for the machining of titanium and nickel-based superalloys for aerospace engineering. Cui et al. [283] manufactured Al_2_O_3_/Ti(C,N) cutting tools containing 0.4 wt.% GNP for continuous dry turning of Inconel 718. This composite showed enhanced mechanical properties and decreased friction between the insert and workpiece on dry reciprocating sliding tests. Based on these results, Inconel 718 was then machined with the composite cutting tool at ultra-high speed using a turning center and dry conditions. The cutting tools exhibited high abrasive wear resistance and better cutting performance than commercial sialon cutting tools, as a direct consequence of the graphene additions.

Regarding lubricated tribological applications, the work of Zhang et al. [284] on 3Y-ZrO_2_/0.15 wt.% GO composites for dental implants should be mentioned. The materials were tribologically tested against GCr15 steel bearings using artificial saliva lubrication to simulate the teeth operating conditions. For that purpose, a reciprocating motion with a frequency of 2 Hz was selected, applying a load of 30 N for reproducing chewing forces. Results indicated that GO additions decreased both the friction coefficient and the wear rate. Besides, the composite was biocompatible and promoted cell proliferation. Balazsi et al. [285] and Zhang et al. [286] analyzed the tribological properties in an aqueous environment of Si_3_N_4_/multilayer graphene (5 wt.%) and SiC/multilayer graphene (2 wt.%) composite pins, respectively, tested against SiC seal rings. In both cases, noticeable reductions of the friction coefficient and wear rate were mutually reported. The water lubrication produced mixed lubrication conditions where the contact pressures decreased and the contact area increased. The water film between the surfaces jointly with the carbon-based tribofilm induced the better tribological response of these composites for seal face components. The tribological performance under isooctane lubrication of different ceramic/graphene composites were chosen to simulate the conditions attained in gasoline direct injection (GDI) engines [4,280]. It is worth mentioning that Si_3_N_4_/GNP composites containing 20.6 vol.% GNP [287] reduced ~50% the friction coefficient of the plain ceramics and enhanced its wear resistance up to 63%, hence standing out as an excellent choice for improving GDI system performance. Considerably reductions in fuel consumption and harmful emissions to the atmosphere were reported, which are of interest to the automotive industry.

### 9.3. Ceramic/Graphene Structures with Damping and Superelastic Performances 

**Figure 23:**
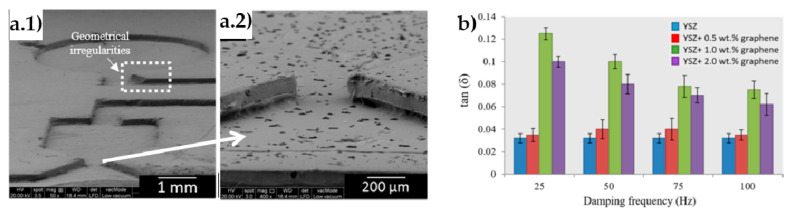
SEM images of micro-thruster section of t-YSZ/GNP composite, showing the overall configuration (**a.1**) and a detail of the micro-nozzle (**a.2**); and (**b**) damping characteristics (tan δ) of the composites and plain YSZ at different frequencies, adapted from [288].

The use of GRM produces very high strength-to-weight ratios in highly porous ceramic/graphene structures, which have unique damping and superelastic responses, and show good prospects for applications in dissipation of impact energy and vibration protection systems, as well as for acoustic insulation [264]. For example, a microthruster was fabricated by gel-casting using zirconia toughened alumina as structural material and a tetragonal YSZ (t-YSZ)/GNP composite as electrode material [288]. The device (Figure 23a) was lightweight (~6.18 g) and able to generate thrust above 100 mN; then, it could be potential primary propulsion for nanosatellites. The addition of 1 wt.% graphene to YSZ not only provided electrical conducting paths (90 S·m^−1^) for electrolytic decomposition but significantly enhanced the damping behavior of the YSZ/GNP composite Figure 23b at low frequencies. 

### 9.4. Structural Health Monitoring (SHM) Uses

SHM sensors are crucial in detecting stress/strain data in diverse structures to avoid overloads that might lead to breakdown or collapse of the entire structure. Picot at al. [289] demonstrated the in-situ self-monitoring capability of ceramic/graphene composites. By combining freeze-cast GO foams with preceramic polymers, and using SPS, a composite with high electrical conductivity (500 S·m^−1^ in the ice growth direction) was produced. The local disruption of the graphene network by crack formation and propagation led to a measurable increase (~0.1 mV) of voltage (constant current) in notched bend bars for very small crack propagations (~10 μm). On the other hand, an improvement of the fiber/matrix interfacial bonding of CF composites used as piezoresistive SHM sensors was achieved by growing GO on the surface of CF, these sensors were tested in aerospace, military and civil engineering use [290,291].

### 9.5. Cement/Graphene Composites

Cement-based composites (CBC) (cement paste, mortar and concrete), the most used materials in construction, display enhanced mechanical properties and damping capability by GRM additions [292,293,294]. The great interest in GRM as an additive for enhancing CBC performance is evidenced by a large number of published works during the last five years, including several reviews [295,296,297]. Key goals in cement and concrete research comprise increasing strength, service life, thermal-energy efficiency, sustainability, and reduce environmental impact. The novel CBC/graphene composites add interesting functionalities such as electrical conductivity and self-sensing potential for a variety of concerns, e.g., vibration measurements, damage detection, SHM, EMI shielding, self-heating pavements for deicing, etc. The preferred GRM for cement is GO because its water dispersibility. In general, the integration of small amounts of GO into a cement matrix produces considerable improvements of the mechanical properties (compressive, flexural, tensile strengths) while induces significant microstructural changes in the cement matrix, affecting the hydration products, promoting pore size reductions and strong matrix/GO interfacial bonding [297,298,299,300,301]. The reported reinforcing effects are variable, depending on the fabrication and mixing methods; for example, compressive strength enhancements in the range of 11–83% have been reported at 28 days for the same GO content (0.02 wt.% respect to the cement) [297]. The addition of GO also improves the corrosion resistance, as these nanostructures seem to block the transport of aggressive agents within the cement matrix and improve the resistance to carbonation, frost and calcium leaching of cement composites.

The main concerns regarding GO nano-reinforcements for CBC are their possible agglomeration, the reduction of the fluidity of the cement paste due to GO water absorption, and the crosslinking of GO with Ca^2+^ ions. Accordingly, modifications of these fillers (e.g., functionalization, coatings and surfactants) have been proposed to moderate these effects and achieve better mechanical performance. Using any of those modifications, varied mechanical enhancements were reported, for example, increases of compressive (~56%) and flexural strengths (77%) for polycarboxylate-modified GO fillers [302], a reinforcement of 133% for silica-coated GO fillers [303], or improvements in flexural (24%) and compressive (32%) strengths for amino-functionalized GO nanosheets [304].

Different graphene sources have been recently proposed to avoid some of the problems associated to GO; besides GNP, some authors have tried electrochemically exfoliated (EE) graphene [305] or multilayered graphene obtained by plasma activation and liquid exfoliation of graphite flakes [306]. EE graphene dispersed well in ordinary Portland cement matrix without the need of surfactants and demonstrated up to 79% increase of tensile strength with just 0.05 wt.% of graphene but with a slight increase of compressive strength (8%). Using EE graphene, other authors evidenced ~35% strength enhancement under compressive and tensile stress tests, for graphene sizes in the range of 50–70 μm, for both short and high curing times (8/28 days) [307]. Even, graphene derived from natural sources as rice-husk has been used, which demonstrated good thermal efficiency in cement composites, compared to other carbon-based materials [308]. GNP additions have led to an enhancement of CBC mechanical properties; for example, increments up to 131% and 95% for splitting tensile and compressive strengths, respectively, at 28 days, have been reported for ordinary Portland cement mortars when using low GNP contents (0.033% by weight of cement) [309] without other additives. 

In addition to the enhanced mechanical performance, CBC/graphene composites can be used for improving the efficiency of energy consumption in buildings, as well as for SHM self-sensing and de-icing applications [310,311,312,313]. For example, the self-sensing characteristics of CBC/graphene composites have been verified for ordinary Portland cement/GNP composites (up to 0.1 wt.% referred to cement) [312] by measuring the electrical conductive changes under loading cycles. 

Some target application areas according to the review paper by Tian et al. [313] are seismic damage detection, self-sensing of concrete for extended nuclear fuel storage systems, weight in motion, smart traffic monitoring, traffic detection, micro-crack detection analysis, corrosion process monitoring and structural health monitoring. The combination of GRM with other conductive particles also led to electrically conductive concrete and mortars, which have been proposed for de-icing applications in roadways and sidewalks through Joule effect heating [314]. 

Pisello et al. [315] investigated optical features, thermal characteristics, electrical properties and strain-sensing capability of CBC composites containing 2 wt.% of different carbon nanofillers, and concluded that GNP was the most effective to increase thermal conductivity (46%), which authors associated to the GNP capability to disperse the thermal wave. This holds great implications on the application of the CBC/GRM composites for radiant paving systems or boreholes concretes in energy plant systems. GNP also produced the largest increase of electrical conductivity (one order of magnitude larger than the plain CBC) and capacitance, and exhibited piezoresistive properties determined as electrical resistance variations under axial deformation, although lower than the measured for MWCNT inclusions. All these carbon nanoinclusions also reduced the solar reflectance capability, while they produced negligible variations in thermal emittance, which should be considered when CBC composites are exposed to outdoor solar radiation, as could increase material overheating due to their solar absorption increase.

Dimov et al. combined graphene with concrete by mixing water-graphene dispersions with ordinary Portland cement, fine dry sand, and 10 mm coarse aggregate [316]. The high-shear exfoliation of graphene in the water proved extremely efficient for the fabrication of graphene-reinforced concrete as it substituted water in the concrete mixture, and the process resulted industrially scalable. This multifunctional nano-engineered concrete showed enhanced properties when compared to standard concrete, in particular, increases of 146% and 79.5% of compressive and flexural strengths, respectively, were achieved, whilst at the same time electrical and thermal performances were improved, the latter mainly through the increase in heat capacity of concrete by the graphene reinforcements. Graphene–concrete composites acted as a barrier against water infiltration, which is an extremely desired property for the long durability of concrete structures, as a matter of fact, the remarkable decrease in water permeability by nearly 400% compared to normal concrete makes this composite ideally suitable for constructions in areas subject to flooding. Besides, graphene inclusions would lead to about a 50% reduction of the required concrete material while still fulfilling construction specifications, which would imply a significant reduction of the carbon emissions by 446 kg per ton linked to cement manufacturing. 

The enhanced damping capability in CBC by GRM additions [292,293,294] has been attributed to the GRM interlayer slip and the viscous friction between GRM and matrix. For example, Long et al. [294] revealed improved dynamic mechanical properties of cement paste/GO, which was linked to internal contact surfaces, porosity, and non-uniform stress distribution. The good shear deformation properties of the GRM network resulted in a high value of its loss factor while the stiff cement matrix ensured a high storage modulus. If the loss factor of GO-containing cement paste could be increased from the original 5 to 10% or better to 15%, its storage modulus would be enhanced without using additional dampers or control devices. 

Regarding energy consumption efficiency, GRM additions to common phase change materials used in cement have been reported [317] to increase the poor thermal conductivity of PCM and avoid leakage phenomena. In particular, GNP additions in a mixture of paraffin (PCM) and hydrophobic expanded perlite produced an increase of the effective thermal conductivity of 49% and also reduced the heat storage/retrieval duration by 20% with just 0.5 wt.% GNP. Convective heat-gain rates of 78% and 125% during heat storage and discharge, respectively, were reported for test room experiments using cement mortars with GNP modified PCM for the walls. In this context, the thermoelectric response of CBC/GNP composites has also been evaluated.

Recently, GO has shown its effectiveness in achieving a cementitious mixture for 3D printing applications. The printable cementitious mixture was fabricated from ordinary Portland cement, industrial waste materials, GO, and chemical additives. GO improved the printability, the fresh mechanical properties and after 28 days curing as well (compression strength of 125 MPa), signaling this route as a viable method for 3D fabrication of a wide range of engineered cementitious materials for civil and construction applications, which is a promising novel manufacturing technology for reducing cost in the use of cement materials [318].

To summarize, GRM can be used to promote construction sustainability with the following potential contributions: less cement consumption, greatly improved mechanical properties, promoting innovative architectural and lighter structural designs, excellent permeability resistance due to low porosity, high early strength that can reduce construction cycle times, energy consumption efficiency and additional interesting functionalities like self-sensing of early damage, damping, de-icing, and even ultra-strong EMI shielding, and fire resistance ability due to the higher thermal conductivity.

## 10. Applications of Additive Manufactured Ceramic/Graphene Composites 

The outcomes in this topic have received a notable impulse in recent years owing to the notable expansion of 3D printing methods and the noteworthy effects of graphene type inclusions in ceramic composites [319,320]. These nanostructures are prized because of their effectiveness as reinforcing agents but also due to the added electrical and thermal functionalities or the specific surface area enhancing effect that commonly provides [4,321]. These characteristics are of paramount interest given the intended applications of these 3D composite structures [322], which comprise catalytic uses, bio-implants, heat dissipation, electrodes in supercapacitors and batteries, sensors, wearable electronics, etc. Additive manufacturing methods include many techniques, such as direct ink writing (DIW), fused deposition modeling, stereolithography, selective laser sintering, ink-jet printing, etc., whose characteristics and advantages have been reviewed in several papers [323,324]. We present below a summary of the most relevant works published recently on 3D printed ceramic-graphene composites, providing a succinct description of the preparation procedure and printing method, and obviously of the applications pursued or tested in each case of composite. 

### 10.1. Si_3_N_4_, SiC Type Composites

3D SiC/GNP composite structures with variable contents of GNP (5–20 vol.%) were processed by DIW from water-based composite inks, displaying electrical conductivity that increased with GNP additions. Directional response of σ_e_ was demonstrated owing to the log-pile design of the 3D structure and the reported GNP alignment during printing. Conversely, the compressive strength of the 3D structures decreased with GNP content due to the observed porosity increase. The importance of rod-to-rod contact resistance in determining the electrical conductivity of these structures was noted using modeling tools that were claimed to be applicable to address other properties of the structures [325]. 

Other authors reported an alternative method for fabricating these composites consisting of DIW of GNP using ethylene glycol butyl ether and ethanol media, followed by CVI with a SiC precursor (methyltrichlorosilane) to form structured composites. As a result, β-SiC grains nucleated between the sheets of the graphene filling the voids and also formed a dense SiC rim on the filaments for the longer CVI times. The electrical conductivity and compressive strength in these composites increased with infiltration time (up to 50 h) and graphene concentration in the inks (25–50 wt.%) reporting maximum values of 3700 S⋅m^−1^ and 200 MPa (50 h), respectively [326].

**Figure 24:**
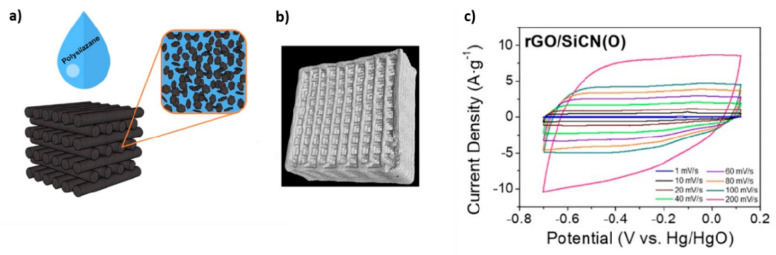
(**a**) Schematics showing fabrication of 3D printed SiCN/rGO structure by impregnation of a DIW rGO with a preceramic polymer; (**b**) tomographic image of the treated structure (800 °C); (**c**) cyclic voltammogram at different scan rates from 1 to 200 mV·s^−1^ with an ideal capacitive behavior. Reprinted from reference) [328], copyright (2019), with permission from Elsevier.

3D composites of rGO and amorphous SiCN were also obtained through infiltration; in this case, GO scaffolds were first printed by DIW using aqueous inks and subsequently immersed in an organic-polysilazane liquid polymer, which after crosslinking and pyrolysis (800–1000 °C) formed the amorphous ceramic phase [327]. The relative matrix content was controlled by diluting the polymer. The structured composite demonstrated high values of electrical conductivity (870 S⋅m^−1^), even at high temperatures, particularly if the GO structure was previously reduced, as well as notable thermal resistance to direct flame and considerable mechanical strength (10 MPa). This kind of 3D composite was tested as supercapacitor electrodes (50.3 F⋅g^−1^ at 0.06 A⋅g^−1^) showing large capacity retention after 7000 cycles Figure 24 [328,329]. 

Using a different perspective, 3D SiOC ceramic structures were printed from preceramic polymers (a methyl silicone resin) with small GO amounts (<1 wt.%) to confer integrity to the structure and reduce contractions of the preceramic polymer during crosslinking and pyrolysis [330]. In other work, GO sheets were used to adjust the ink rheology for achieving proper viscoelastic characteristics of the inks for DIW, instead of employing polyelectrolytes, in particular, this line was tried for alumina platelets and SiC powders (GO contents < 4 vol.%) [331].

### 10.2. Alumina, Silica Composites 

Structured composites of Al_2_O_3_ with GO (0.5–5 wt.%) additions were prepared by DIW from a mix of colloidal ink of different polyelectrolytes. The structures, treated at 1500 °C in Ar/H_2_ atmosphere for consolidation and reduction, proved their effectiveness as metal-free catalysts in various Paal-Knorr model reactions to synthesize substituted pyrroles, while maintained their structural integrity and the possibility of regeneration after use [332].

By adding alumina powders (up to 70 wt.%) and GNP (maximum of 4 wt.%) to biodegradable polylactic acid (PLA) and hot pressing, researchers developed PLA-alumina-graphene composites with enhanced thermal conductivity (up to 2.1 Wm^−1^ K^−1^) with respect to the PLA alone (~0.5 Wm^−1^ K^−1^). These composites were cryogenic milled and mixed with fumed silica for allowing processing by selective laser sintering into complex shapes with the aim of building enhanced heat dissipation devices for the electronic industry [333]. 

The development of 3D rGO scaffolds grafted with silica *np* was reported by using sol-gel and DIW methods. The method also proved effective for Al-Si sols forming in that case a silicoluminate *np*/rGO hybrid scaffolds. First, GO structures were printed, freeze-dried and reduced by thermal treatment. The structures were then immersed in the corresponding sol and later subjected to ammonia vapors for condensation of the gel. The attached silica (or silicoaluminate) xerogel notably enhanced the compressive strength of the rGO scaffolds (in the range 100–400 kPa), inducing a considerable capacity for water absorption as well, thus expecting their potential applications as absorber and pollutant removal [334], in all cases the structures remained electrically conductive as well.

### 10.3. Bioceramic Scaffolds

On the subject of biomaterials for bone tissue engineering applications, we can mention the use of DIW to create 3D mixed composites of the biodegradable polylactic-co-glycolic acid (PLGA) copolymer with hydroxyapatite powders and GNP (10–90 vol.% referred to HA), with maximum polymer contents of 10–40 vol.%, which solidify rapidly by the use of a convenient mixture of three solvents. These scaffolds were used for in vitro studies through mesenchymal stem cell seeding, reporting cell proliferation, although a clear neurogenic effect assumed by the conducting character of the scaffolds was not observed [335]. In a different work, researchers created HA scaffolds with small additions of graphene (0.5–1 wt.%) with the aim of developing a photothermal effect in the composites that could be effective for BTE by promoting osteogenesis through local heating. Chitosan and gelatin were used as biodegradable binders and glutaraldehyde acted as a cross-linker. Enhanced heating was observed for the graphene-containing scaffolds after short exposure to laser radiation in the near-infrared region, which also produced increased cell proliferation [336].

The printing of bioactive glass composition (45S5 Bioglass^®^) with different amounts of rGO (0.5, 1, 1.5, 2, and 3 vol.%) by DIW was reported using water as a solvent and carboxymethyl cellulose as a viscosity modifier, results indicated that optimal mechanical enhancement was achieved for the 1 vol.% rGO composite sintered at 500 °C [237]. Within the bone graft materials for in bone surgery applications, we can also highlight the development of tricalcium phosphate (β-TCP)-GO scaffolds modified with Fe_2_O_3_, looking in this case for a magnetic functionality usable in hyperthermia therapy. Accordingly, β-TCP scaffolds were DIW using aqueous mixtures with polyvinyl alcohol, which were sintered at 1100 °C once conveniently dried. The scaffolds were subsequently immersed in Fe_2_O_3_-GO and GO solutions, repeating this process several times to achieve a layered Fe_2_O_3_-GO/GO microstructure on the β-TCP filaments. Results indicated that the magnetothermal effect induced in the scaffolds actually killed tumor cells and stimulated osteointegration as well [337].

### 10.4. Clay, Geopolymer Type Composites

**Figure 25:**
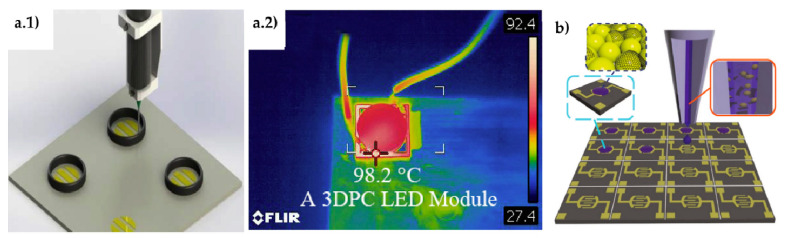
(**a.1**) Printing of a kaolin/rGO cavity; (**a.2**) infrared camera images of a LED module within the 3D printed cavity reprinted from reference [340], copyright (2020), with permission from Elsevier; (**b**) Illustration of an inkjet deposition process on a CMOS platform of a ZnO/graphene mix for a sensory device, reprinted from reference [341].

Some studies have shown the possibility of extrusion printing mixtures of geopolymers (typically alkali silicates and aluminosilicates) and GO (contents from 5–20 vol.%) plus water with no other additives. The GO addition definitively made the geopolymer printable by DIW methods. The printed structures needed to set at a fixed temperature (5 °C) and humidity conditions prior to sintering at temperatures of 1000 °C, rendering conductive robust structures [338]. A similar route was followed to make 3D composites of kaolin and sodium silicate with GO; in this case, the structures obtained by DIW were let to set for several days, thus avoiding any thermal treatment, and results also evidenced the reinforced benefits of the GO addition [339]. A very similar procedure (ink containing clay, sodium silicate and GO plus a titanate coupling agent) was followed to achieve a 3D printed cavity based on a kaolin/rGO mixture for improving heat dissipation in a high-power light-emitting diode electronic module, Figure 25(a.1,a.2), while maintaining the optical properties and airtightness of the LED module [340]. 

Nanoclay particles of laponite and GO sheets have been used to enhance printability and photothermal activity of the responsive hydrogel precursor N-isopropylacrylamide (NIPAAm). A small amount of a photoinitiator was also added to the mixture. The suspensions were kept for one day in dark sealed containers before been ready for DIW. Researchers achieved multi-responsive hydrogels (to heat or light exposures), and predictable undulating shapes in the composites by using two dispensing pumps with slightly different compositions. Combinations of these composite hydrogels were employed to fabricate a microfluidic system feasible for biological and chemical uses [342].

### 10.5. Metal Oxide, Dichalcogenide Type Materials

Several studies have recently been published on printed ZnO-graphene hybrids. We can mention first the inkjet printing of a mixture of ZnO powders and graphene flakes, using N-methyl-pyrrolidone (NMP) as a solvent, on micro-ridged PDMS substrate to achieve a highly stretchable and flexible strain sensor suitable for body sensing. The highest sensitivity for different strain levels was reported for the 1graphene:0.5ZnO ratio [343]. In a different work, researchers used a ZnO sol-gel precursor that was mixed with GNP and deposited by inkjet printing on a heated Si/SiO_2_ substrate (50 °C) to favor capillary bridging. The films were treated at 350 °C to nucleate ZnO and form multiple ZnO/GNP junctions; after testing for ultraviolet light (340 nm) detection, they proved better response than alike composites processed by other methods [344]. On the other hand, mix inks of ZnO nanopowders and graphene were prepared in two alcohols and polyvinylpyrrolidone media for inkjet printing films onto miniaturized complementary metal-oxide-semiconductor (CMOS) micro-hotplates to build portable sensors for breath diagnostics (Figure 25b). The sensor was tested for NH_3_ detection with a reported enhanced overall performance compared to other sensors, with claimed repeatability and scalability based on the CMOS platform used [341]. The same rationale was also applied for WO_3_-graphene hybrids alleging the plausible application to other metal oxide sensing materials.

Within the same ZnO *np*/graphene system, researchers have fabricated a porous photodetector film by inkjet printing taking advantage of the different volatility of co-solvents and the change in surface tension by the graphene presence to create controlled microporosity in the films. The I_light_/I_dark_ ratio of the composite films although small it proved larger than that of the pure ZnO film, and increased with porosity as well [345]. 

Fe_2_O_3_-GNP were extrusion printed using a mixture of polymers, solvents and some water, this mixture settled at RT. The scaffolds were intended for applications in electronic devices, which researchers proved building different gadgets such as a magnet-driven toy car, a magnetic switch, or an electromagnetic interference shielding grit [346].

In the subject of electronic energy storage, efforts have been done for increasing the current density of supercapacitors; for that purpose, Mn_3_O_4_ nanosheets have been mixed with rGO to improve the electric conductivity of the oxide. The composites were achieved by reaction and precipitation of Mn salt and GO mixtures, and subsequent hydrothermal reduction. The composites showed higher specific capacitance than the counterparts and offered the possibility of been shaped by DIW methods [347]. 

For achieving high-performance electrochemical devices some researchers have designed a hybrid system of a bimetallic nickel–cobalt sulfide (Ni–Co–S) and graphene with an architected structure achieved by DIW, aiming at increasing both, the accessibility to active redox sites and the pathways for the ion/electron transfer. To achieve printability characteristics, the precursors (NiCo synthesis product/GO) were mixed with sodium alginate to gain printability. The structures after freeze-drying, reduction and sulfurization, showed a highly interconnected microstructure and large capacity retention. As proof of concept a fully printed device for electrochemical energy storage was assembled (both electrodes) [348].

**Figure 26:**
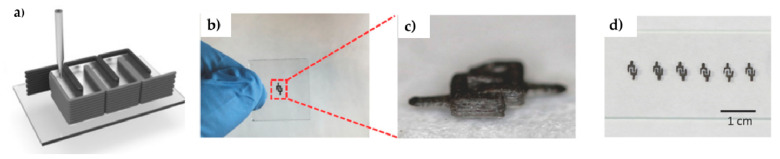
(**a**) Illustration of the printing of the Li-ion battery cell (LTO/GO anode, LFP/GO cathode and electrolyte); (**b**–**d**) images of the actual miniaturized cells reprinted from reference [349], copyright (2016), with permission of John Wiley and Sons, Inc.

In the field of Li-ion batteries, it should be recognized the achievement of an interdigitated battery cell by printing of the anode and cathode using mixtures of GO with lithium titanium oxide (Li_4_Ti_5_O_12_, LTO) and lithium iron phosphate (LiFePO_4_, LFP), respectively. Both electrodes were consecutively printed by DIW on a glass substrate, freeze-dried and annealed at 600 °C in Ar/H_2_ for GO reduction, and accordingly tested in a planar battery electrochemical cell demonstrating high stability. The possibility of a miniaturized design of the cells was demonstrated in Figure 26 [349]. Printing offers the advantage of interdigitated designs compared to more conventional methods. 

Regarding the future Na ion batteries, an attempt has been reported on the printing of a porous MoS_2_/GO anode intended to accommodate the larger Na ions and larger volumes changes during the charge/discharge cycles. The method chosen was inkjet printing of a mixed ink of GO and a MoS_2_ precursor in combination with the freezing of the printed drops by cooling at −30 °C the substrate, later freeze-drying and annealing at 600 °C in Ar/H_2_ for GO reduction and preserving the porous nature. The electrochemical characterization indicated the potential of this 3D printed MoS_2_/rGO porous electrode for Na ion batteries [350].

## 11. Prospective

Ceramic/graphene composites and hybrids are attractive alternatives for the fabrication of more efficient, lighter and compact batteries. Product commercialization will be a reality once relevant issues for large-scale production, i.e., facile and standardized synthesis routes, control of graphene agglomeration and reproducibility of homogeneous components, are solved. Equally, supercapacitor fabrication is one of the fields in which the whole potential of GRM composites can be successfully exploited in the near term. Further efforts are needed for application in electrodes for fuel cells to gain more knowledge on long operation times. In the case of solar cells, the actual development is at a research stage for a better understanding of the effects on charge transport mechanisms.

Ceramic/graphene materials have shown good responses in energy harvesting but need to be combined with specific product designs to maximize efficiency. Nano and micro-generators are clean novel technologies but still in an early development stage, hence, more efforts will be necessary for the following years. These materials will be relevant for future portable and wearable devices.

Graphene-based compounds and hybrids have clearly demonstrated improved overall performance as electrochemical sensors and biosensors for the detection of molecules biomolecules, bacteria and viruses, which has been the impetus for extensive research in this area. The benefits related to miniaturization and portability are also important goals. Control over manufacturing methods, scalability, and reliability are issues that need to be solved. The development of laboratory-scale detection devices has proven its feasibility and at the moment, market product development is a closer challenge, driven by the current global health juncture and also by crucial environmental issues.

Despite the excellent results of ceramic/graphene composites in biomedicine, in-vivo experiments on the potential toxicity and possible residues are still required. Complex systems, in which osteogenesis-inducing drugs, peptides, proteins, and anti-bacterial agents are incorporated, emerge with great potential in the near future. 

Regarding engineering applications, we can mention the excellent tribological results attained at the lab scale for ceramic/graphene composites that will allow short-term testing in a number of real applications, particularly in cutting and machining operations, engine components, seal face parts and dental implants. The favorable effects of graphene on the short-crack and long-crack toughness, elasticity, strength and dynamic mechanical performance of ceramic materials opens an important area of scarcely explored applications, like impact and ballistic protection, vibration protection, damping and stress/strain self-monitoring. Research in this field is currently focused on cementitious materials with the first graphene reinforced concrete commercial product just announced. An increased effort is expected in the near future for high added-value applications of high-temperature ceramics. GRM has proved to be ideal nanofillers for tuning the heat transfer performance of ceramics. Investigation on the thermal performance of highly porous hierarchical multi-material structures at working conditions, including modeling, will be necessary for the applications of these composites in thermal management.

EMI shielding is one of the most promising applications of ceramic/graphene composites as EMI pollution is an unsolved relevant issue for devices working at high temperatures and harsh environments. Targets in this subject include the development of hierarchical multicomponent structures using PDC and sol-gel precursor technologies. 

The good catalytic performance of both metal-free and metal-containing ceramic/graphene composites would favor new developments for efficient removal of larger number of chemical contaminants and also lead to a higher yield in hydrogen production in the next years.

The outlook for additive manufacturing methods in relation to the industrial applications of ceramic/graphene composites is brilliant as they offer advantages such as precise computerized designs, miniaturization and multi-material 3D structures. Reductions of waste and toxic materials are opportunities of additive manufacturing methods in addition to the advantages of improved flexibility and integration, compared to other technologies. Particularly promising are the applications as bio-materials scaffolds, supercapacitors, batteries and sensors. 

Summarizing the application prospects for ceramic/graphene composites show varying degrees of development, as they include many different areas, some of them look quite real but future developments are very dependent on the availability of standardized GRM sources, the fair control over manufacturing methods, scalability and reliability, but also on external factors such as societal challenges, environmental and health issues.

## Data Availability

Not applicable.
